# Clinical and molecular characterization of 40 patients with classic Ehlers–Danlos syndrome: identification of 18 *COL5A1* and 2 *COL5A2* novel mutations

**DOI:** 10.1186/1750-1172-8-58

**Published:** 2013-04-12

**Authors:** Marco Ritelli, Chiara Dordoni, Marina Venturini, Nicola Chiarelli, Stefano Quinzani, Michele Traversa, Nicoletta Zoppi, Annalisa Vascellaro, Anita Wischmeijer, Emanuela Manfredini, Livia Garavelli, Piergiacomo Calzavara-Pinton, Marina Colombi

**Affiliations:** 1 Division of Biology and Genetics, Department of Molecular and Translational Medicine, University of Brescia, Viale Europa 11, 25123 Brescia, Italy; 2 Department of Dermatology, University Hospital Spedali Civili, Brescia, Italy; 3 Clinical Genetics Unit, Istituto di Ricovero e Cura a Carattere Scientifico, Arcispedale S. Maria Nuova, Reggio Emilia, Italy; 4 Department of Medical Genetics, Policlinico Sant’Orsola-Malpighi, University of Bologna, Bologna, Italy; 5 Dipartimento Materno Infantile, Ospedale Niguarda Ca’ Granda, Milan, Italy

**Keywords:** Classic Ehlers–Danlos syndrome, *COL5A1*, *COL5A2*, *COL1A1*, MLPA, Diagnostic flowchart

## Abstract

**Background:**

Classic Ehlers–Danlos syndrome (cEDS) is a rare autosomal dominant connective tissue disorder that is primarily characterized by skin hyperextensibility, abnormal wound healing/atrophic scars, and joint hypermobility. A recent study demonstrated that more than 90% of patients who satisfy all of these major criteria harbor a type V collagen (COLLV) defect.

**Methods:**

This cohort included 40 patients with cEDS who were clinically diagnosed according to the Villefranche nosology. The flowchart that was adopted for mutation detection consisted of sequencing the *COL5A1* gene and, if no mutation was detected, *COL5A2* analysis. In the negative patients the presence of large genomic rearrangements in *COL5A1* was investigated using MLPA, and positive results were confirmed via SNP-array analysis.

**Results:**

We report the clinical and molecular characterization of 40 patients from 28 families, consisting of 14 pediatric patients and 26 adults. A family history of cEDS was present in 9 patients. The majority of the patients fulfilled all the major diagnostic criteria for cEDS; atrophic scars were absent in 2 females, skin hyperextensibility was not detected in a male and joint hypermobility was negative in 8 patients (20% of the entire cohort). Wide inter- and intra-familial phenotypic heterogeneity was observed. We identified causal mutations with a detection rate of approximately 93%. In 25/28 probands, *COL5A1* or *COL5A2* mutations were detected. Twenty-one mutations were in the *COL5A1* gene, 18 of which were novel (2 recurrent). Of these, 16 mutations led to nonsense-mediated mRNA decay (NMD) and to COLLV haploinsufficiency and 5 mutations were structural. Two novel *COL5A2* splice mutations were detected in patients with the most severe phenotypes. The known p. (Arg312Cys) mutation in the *COL1A1* gene was identified in one patient with vascular-like cEDS.

**Conclusions:**

Our findings highlight that the three major criteria for cEDS are useful and sufficient for cEDS clinical diagnosis in the large majority of the patients. The borderline patients for whom these criteria fail can be diagnosed when minor signs of connective tissue diseases and family history are present and when genetic testing reveals a defect in COLLV. Our data also confirm that *COL5A1* and *COL5A2* are the major, if not the only, genes involved in cEDS.

## Background

Classic Ehlers–Danlos syndrome (cEDS) (MIM #130000) is a rare autosomal dominant disorder with a prevalence of 1/20,000 and is characterized by skin hyperextensibility, widened atrophic scars, and generalized joint hypermobility, which are the major criteria of the Villefranche nosology [1,2]. Joint hypermobility is assessed according to the Beighton scale [2]. Other prominent features of cEDS include smooth, velvety skin, molluscoid pseudotumors, subcutaneous spheroids, complications of joint hypermobility (e.g., sprains, dislocations/subluxations, *pes planus*), muscle hypotonia, delayed gross motor development, easy bruising, manifestations of tissue extensibility and fragility (e.g., hiatal hernia, mitral valve prolapse, anal prolapse in childhood, cervical insufficiency, rectal and uterine prolapse), surgical complications (postoperative hernias), and positive family history. The clinical manifestations range in severity, and families with mild to severe expression have been described [1-6].

Muscle hypotonia and delayed gross motor development can be early presentations of the disorder, as can uni- or bi-lateral hip dislocations. Certain adults also suffer from chronic musculoskeletal pain. Pregnancy and delivery may present complications, especially in the more severe forms. Premature rupture of membranes can occur, and there is an increased risk for extension of the episiotomy, tearing of the perineal skin, and pelvic prolapses. Aortic ring dilatation, large-size arterial aneurysms and dissections, intracranial aneurysms, and arterio-venous fistulas have rarely been observed [3-9].

There is a significant clinical overlap of cEDS with other EDS variants, especially hEDS. In hEDS, generalized joint hypermobility with complications, such as repetitive dislocations and chronic articular pain, are the primary clinical manifestations. In this condition, the skin abnormalities are generally subtler than in cEDS given that the skin is generally poorly extensible, and atrophic scars present post-surgically [1,10, our unpublished results].

The molecular basis of cEDS is essentially a deficiency of type V collagen (COLLV), which is a quantitatively minor fibrillar collagen that is widely distributed in a variety of connective tissues [11]. The major variant of COLLV is a heterotrimer that is composed of two pro-α1(V) chains and a single pro-α2(V) chain, wich are encoded by the *COL5A1* and *COL5A2* genes, respectively. COLLV plays a central role in collagen fibrillogenesis and co-assembles with type I collagen (COLLI) to form heterotypic fibrils. The entire triple helical domain of COLLV is buried within the heterotypic fibril, and only the N-terminal NH2-propeptide is exposed at the surface [12,13]. The NH2-propeptide includes the α1(V)-N-propeptide, which is composed of four domains: the N-terminal thrombospondin-1-like (TSPN-1), the variable (VAR) domain, the small interrupted collagenous (COL2) and non-collagenous (NC2) domains, and the α2(V)-N-propeptide, which consists of one cysteine-rich domain (CR). Following proteolytic processing by Bone Morphogenetic Protein-1 and furin, the mature α1(V)-chain retains the VAR, COL2, and NC2 domains; the mature α2(V)-chain retains the CR domain [14]. The NH2-propeptide has a key regulatory function in fibril assembly. This propeptide regulates the diameter of the heterotypic I/V collagen fibrils and is essential for interactions of COLLV with the other components that are involved in the maintenance of ECM architecture [12-16]. Ultrastructural examination of skin from patients with cEDS reveals irregular and loosely packed collagen fibrils and the presence of typical “cauliflower” fibrils, which represent the histological hallmark of the disturbed fibrillogenesis of the heterotypic collagen fibrils [17].

Recently, a comprehensive molecular study demonstrated that over 90% of the patients strictly satisfy the three major criteria for cEDS and harbor a COLLV defect [18]. The most recent update of the LOVD Ehlers–Danlos Syndrome Variant Database lists 117 different mutations that affect COLLV. Specifically, 100 distinct *COL5A1* (approximately 85%) and 17 *COL5A2* mutations are described (http://www.le.ac.uk/genetics/collagen/, November 2012, [19]). Whereas *COL5A1* mutations are scattered throughout the gene, all of the *COL5A2* mutations are located within the triple helix domain, except for one mutation in the C-propeptide [4]. Defects in *COL5A1* are most commonly null mutations, consisting of either nonsense, frameshift or splice site mutations that introduce a premature termination codon (PTC). This latter type of mutation generates an unstable transcript that is rapidly degraded by NMD and results in an overall reduction in COLLV levels (i.e., haploinsufficiency) [4,8,18,20-27]. Only a small number of missense or in-frame exon-skipping splice mutations are described for *COL5A1*[4,9,18,28,29]; in contrast, nearly all of the *COL5A2* mutations reported to date represent missense or in-frame exon-skipping splice mutations, which result in the production of mutant α2(V)-chains that are expected to incorporate into COLLV molecules [4,18,25,26,30-32]. Although the majority of *COL5A1* mutations are null mutations, certain structural *COL5A1* mutations can reduce the amount of normal COLLV that is available in the ECM. Specifically, leucine substitutions in the signal peptide domain of the pre-pro-α1(V)-chain were demonstrated to impair the secretion of mutant COLLV [4,33]. Moreover, mutations that result in the deletion of a highly conserved cysteine in the C-terminal propeptide prevent the incorporation of the mutant pro-α1(V) chains into the molecule [18,21,26]. A notable type of *COL5A1* mutations is represented by 3 splicing errors within the highly conserved α1(V)-N-propeptide. These errors result in several in-frame splice transcripts that are expressed and secreted into the extracellular environment [18,34,35]. The precise mode of action of these N-propeptide mutations is less well understood, as are the triple helix substitutions that result from either *COL5A1* or *COL5A2* mutations. These mutations are predicted to result in the production of mutant α1(V) or α2(V)-collagen chains that interfere with the formation of COLLV heterotrimers. In turn, secreted mutant heterotrimers are predicted to exert a dominant negative effect, perturbing the heterotypic fibril formation and the interactions of COLLV with other ECM constituents [4,16,18,32-35].

To date, no clear genotype-phenotype correlations for COLLV defects in patients with cEDS have emerged. Although the number of patients with a *COL5A2* mutation is limited compared to the number of patients who harbor a *COL5A1* defect, recently published results indicate that *COL5A2* mutation carriers fall within the more severe end of the phenotypic spectrum of cEDS [18]. Certain patients with a mild presentation are also reported but a detailed clinical presentation is lacking [4,18,31,32].

Mutations in certain other connective tissue genes, such as *COL1A1* and *TNXB*, have been described to cause phenotypes that strongly overlap with cEDS. However, these mutations appear to be very rare, especially *TNXB* mutations. Moreover, the associated phenotypes exhibit certain differences from those of COLLV-associated cEDS [11,36,37]. Specifically, missense mutations in *COL1A1* that result in the substitution of an arginine residue in the Xaa position of the Gly-Xaa-Yaa repeat with a cysteine, causing a dominant negative effect, cause a cEDS phenotype with a propensity for arterial rupture in young adulthood (i.e., vascular-like cEDS) [3,6,11,36,38].

Here, we report the clinical and molecular findings for 40 patients with cEDS from 28 families. The present results support the recently published data by Symoens et al. [18], confirming that *COL5A1* and *COL5A2* mutations are responsible for more than 90% of cEDS cases. In this cohort, we demonstrate that the three major diagnostic criteria are effective in reaching a clinical diagnosis in the majority of such patients.

## Materials and methods

### Patients

All of the subjects provided written, informed consent and authorized the processing of their personal data according to Italian bioethics laws and the Declaration of Helsinki Principles were respected. The cohort included 38 patients of Italian origin and two children, one from Morocco and the other from Moldavia, who were referred to the Genetics Service of the University of Brescia and to the Center for Heritable Connective tissue disorders and Ehlers–Danlos syndrome at the Dermatology Department of the Spedali Civili of Brescia. All of the patients were recruited and examined both clinically and at the molecular level within the past 3 years. Detailed clinical records and family histories were obtained for all of the patients according to the Villefranche nosology. The presence of the three major criteria for cEDS, i.e., skin hyperextensibility, widened atrophic scars, and generalized joint hypermobility, was evaluated. The presence of the minor cEDS criteria was also ascertained, including the following: a history of delayed gross motor development; dysmorphic signs, e.g., blue sclerae, micro-retrognathia, epicanthal folds, and high palate, etc.; dermatological signs, i.e., smooth and velvety skin, easy bruising, molluscoid pseudotumors, piezogenic papules, *striae distensae*; articular and skeletal signs, i.e., *pectus excavatum*, valgus knee, *pes planus*, *hallux valgus*, kypho/scoliosis, joint dislocations/subdislocations, and chronic articular pain; cardiovascular signs, i.e., valvular regurgitation, mitral valve prolapse; and other features, e.g., chronic fatigue syndrome and gastrointestinal involvement (Table 1) [2,3,6,18].

**Table 1:** Clinical findings of the 40 patients with cEDS

**Patient IDs**	**Sex/Age (y)**	**Family history**	**Hyperextensible smooth skin**	**Scars**	**Joint hypermobility Beighton score**	**Other features**
**AN_002501**	**F/15**	**-**	**+**	**+ A**	**+ (9/9)**	**Easy bruising, *Striae distensae*, Piezogenic papules, *Pes planus*, Dislocations, Chronic articular pain**
**AN_002502**	**F/29**	**-**	**+**	**+ A**	**+ (7/9)**	**Hypotonia at birth, Delayed motor development, Blue sclerae**^**a**^, **Hypertelorism, Epicanthus, Micrognathia, Hypermetropia, Astigmatism, Reduced anterior chamber depth, Easy bruising, Piezogenic papules, Palmar creases, Hands and feet deformity, Scoliosis, Spondylolisthesis, *Hallux valgus*, *Pes planus*, Renal ptosis, Gastroesophageal reflux**
**AN_002503 father of**	**M/40**	**+**	**+**	**+ A**	**+ (9/9)**	**Thin lips, Easy bruising, Molluscoid pseudotumor (elbow), Piezogenic papules, *Pes planus*, Varicose veins**
**AN_002504 sister of**	**F/13**	**+**	**+**	**+ A (one)**	**+ (9/9)**	**Epicanthus, Micrognathia, Easy bruising, Piezogenic papules, *Pes planus***
** AN_002505**	**F/6**	**+**	**+ Doughy**	**+ A, H**	**+ (9/9)**	**Epicanthus, Micrognathia, Easy bruising, Piezogenic papules, *Pes planus***
**AN_002506**	**M/24**	**-**	**+ Doughy**	**+ A**	**+ (5/9)**	**Bleeding gums, Dysphonia, Myopia, Easy bruising, Piezogenic papules, *Hallux valgus*, *Pes planus*, Osteoporosis, Dislocations (recurrent), Severe chronic articular pain (daily therapy with FANS), Aortic regurgitation, Mitral valve prolapse, Inguinal hernia, Chronic fatigue syndrome**
**AN_002507 daughter of**	**F/10**	**+**	**+ Doughy**	**+ A**	**+ (9/9)**	**High palate, Mild prognathism, Easy bruising, Dental malocclusion treated with byte**
**AN_002508**	**F/40**	**+**	**+ Doughy**	**-**	**+ (5/9)**	**Blue sclerae, *Striae distensae*, Easy bruising, Mild scoliosis, Transitional lumbosacral vertebra, Subdislocations (sporadic), Low back pain, Chronic sinusitis, Atopy, Hashimoto’s thyroiditis, Melanoma treated with surgery**
**AN_002509**	**M/5**	**-**	**+ Doughy**	**+ A**	**+ (8/9)**	**Blue sclerae, Mild hypertelorism, Micrognathia, Low-set ears, Easy bruising, Piezogenic papules, Valgus knee, Mobile testis, Atopic dermatitis**
**AN_002510**	**M/4**	**-**	**+ Doughy**	**+ A**	**+ (9/9)**	**Delayed motor development, Blue sclerae, Easy bruising, Piezogenic papules, *Pes planus*, Gastroesophageal reflux, Umbilical hernia**
**AN_002511**	**F/15**	**-**	**+ Doughy**	**+ A (one)**	**+ (8/9)**	**Blue sclerae, Easy bruising, Arachnodactyly, Mild scoliosis, Valgus knees, *Pes valgus *and *cavus*, Subdislocations, Syonvitis (hip), Chronic articular pain, Posterior cerebral artery hypoplasia, Anal fissures**
**AN_002512 mother of**	**F/41**	**+**	**+**	**-**	**- (2/9)**	**Blue sclerae, Epicanthus, High palate, Myopia, Easy bruising, *Striae distensae*, Piezogenic papules, *Hallux valgus*, *Pes planus*, Chronic fatigue syndrome**
**AN_002513**	**M/10**	**+**	**+ Doughy**	**+ A**	**+ (8/9)**	**Blue sclerae, Epicanthus, Myopia, Mild strabismus, Micro/retrognathia, High palate, Easy bruising, Piezogenic papules, *Pectus excavatum*, Winged scapulae, Kyphosis, *Pes planus*, Mitral valve prolapse, Tricuspid and pulmonary regurgitation, Atrio-ventricular block (1**^**st **^**and 2**^**nd **^**degree), Umbilical hernia treated with surgery, Chronic fatigue syndrome**
**AN_002514**	**F/34**	**-**	**+**	**+ A**	**+ (5/9)**	**Easy bruising, Plantar creases, Scoliosis, Sacroiliitis, Subdislocations, Chronic articular pain, Right pulmonary artery hypoplasia, Spontaneous abortion (12 w)**
**AN_002515**	**F/7**	**-**	**+ Doughy**	**+ A**	**+ (7/9)**	**Epicanthus, Hypertelorism, Micrognathia, Easy bruising, *Hallux valgus*, Mobile patella, Mild scoliosis, *Pes planus***
**AN_002516**	**F/67**	**-**	**+ Loose**	**+ A**	**+ (8/9)**	**Easy bruising, Deep venous thrombosis, Hypertension, Menometrorrhagia and hysterectomy, Multiple myeloma and death for renal failure (67 y)**
**AN_002517**	**F/33**	**-**	**+ Doughy, Thin**	**+ A**	**- (2/9)**	**Blue sclerae, Astigmatism, Palmar creases, Easy bruising, Piezogenic papules, Mild scoliosis, *Pes planus*, Cervical disc hernias with articular blocks, Subdislocations (sporadic), Chronic articular pain, Osteopenia, Muscle hypotonia, Acrocyanosis, Mild mitral, tricuspid and pulmonary regurgitation, Menometrorrhagia, Gastroesophageal reflux, Periodontitis**
**AN_002518 mother of**	**F/37**	**+**	**+ Doughy**	**+ A**	**+ (9/9)**	**High palate, Low set ears, Micrognathia, Easy bruising, Piezogenic papules, Mild scoliosis, Subdislocations, Discal hernias, Chronic articular pain, Constipation**
**AN_002519**	**M/5**	**+**	**+ Doughy**	**+ A**	**+ (9/9)**	**High palate, Epicanthus, Micrognathia, Easy bruising, Piezogenic papules, Winged scapulae, *Pes planus***
**AN_002520 sister of**	**F/9**	**+**	**+ Doughy**	**+ A**	**+ (5/9)**	**Hypotonia at birth, Blue sclerae, Micrognathia, Myopia, Easy bruising, Dislocations, Chronic articular pain (knees)**
**AN_002521 son of**	**M/7**	**+**	**+ Doughy**	**+ A**	**+ (5/9)**	**Hypotonia at birth, Blue sclerae, Micrognathia, Easy bruising, Dislocations**
**AN_002522 son of**	**M/35**	**+**	**+**	**+ A**	**- (3/9)**	**Blue sclerae, Micrognathia, Easy bruising, Dislocations**
**AN_002523**	**F/60**	**+**	**+ Loose**	**+ A**	**- (3/9)**	**Blue sclerae, Easy bruising, Polyabortivity, Dislocations, Chronic pain**
**AN_002524**	**F/21**	**-**	**+ Doughy**	**+ A, H**	**+ (7/9)**	**Blue sclerae, Epicanthus, Micrognathia, Anteverted nostrils, Easy bruising, Chronic headache**
**AN_002525**	**M/7**	**-**	**+ Doughy**	**+ A**	**+ (7/9)**	**Blue sclerae, High palate, Mild epicanthus, Easy bruising, Piezogenic papules, Valgus knee, *Hallux valgus*, Mobile patella, *Pes planus*, Dislocations (sporadic)**
**AN_002526**	**M/46**	**-**	**+ Doughy**	**+ A**	**- (4/9)**	**Blue sclerae, High palate, Micro-retrognathia, Easy bruising, Onychodystrophy, Piezogenic papules, Mild scoliosis, Spondylolisthesis, Severe bilateral hallux valgus, *Pes cavus*, Dislocations (recurrent), Chronic and generalized pain, Arthrosis, Tendinopathy, Carpal tunnel syndrome, Chronic fatigue syndrome, Hypertension, Diabetes mellitus type II with retinopathy, Recurrent caries, Cataract**
**AN_002527**	**M/48**	**-**	**+ Doughy**	**+ A**	**+ (9/9)**	**Easy bruising, Palmar creases, Mild scoliosis, Arthrosis, Gastroesophageal reflux, Periodontitis**
**AN_002528 twin of**	**F/36**	**-**	**+ Doughy**	**+ A**	**+ (7/9)**	**Hypotelorism, Myopia, Easy bruising, Piezogenic papules, *Pectus excavatum*, Mild scoliosis, *Pes planus*, Subdislocations, Chronic articular pain, Osteopenia, Aortic, tricuspid and pulmonary regurgitation, Mitral valve prolapse, Polyabortivity, Amenorrhea (high prolactin), Hereditary thrombophilia**
**AN_002529**	**F/36**	**-**	**+ Doughy**	**+ A**	**+ (8/9)**	**Hypotelorism, Myopia, Easy bruising, Piezogenic papules, *Pectus excavatum*, Mild scoliosis, *Pes planus*, Subdislocations, Chronic articular pain, Osteopenia, Aortic, tricuspid and pulmonary regurgitation, Mitral valve prolapse, Polyabortivity, Hereditary thrombophilia**
**AN_002530 mother of**	**F/43**	**+**	**+ Doughy**	**+ A**	**+ (9/9)**	**Easy bruising, *Talipes equinovarus *treated with surgery, Mild scoliosis, Hypoplasia of upper femur, *Coxa valga*, Hip prosthesis, Arthrosis, Subdislocations, Chronic articular pain (daily therapy), Tricuspid regurgitation, Gastroesophageal reflux, Chronic gastritis, Polyabortivity, Cervical dysplasia (CIN2) treated with surgery, Atopy**
**AN_002531**	**M/9**	**+**	**+ Doughy**	**+ A, H**	**+ (7/9)**	**Epicanthus, Easy bruising, Piezogenic papules, *Talipes equinovarus *treated with surgery, Mild scoliosis, *Pes planus *and valgus, Tachycardia, Gastroesophageal reflux**
**AN_002532**	**F/7**	**-**	**+**	**+ A, H**	**- (4/9)**	**Epicanthus, Blue sclerae, High palate, Micrognathia, Easy bruising**
**AN_002533**	**M/21**	**-**	**+**	**+ A**	**+ (6/9)**	**Epicanthus, High palate, Strabismus, Hypermetropia, Anteverted nostrils, Micro-retrognathia, Easy bruising, Molluscoid pseudotumor, Mild *pectus excavatum*, Scoliosis, Ulnar deviation, Asymmetric legs, Hands and feet and deformity, *Hallux valgus*, *Pes planus*, Enthesopathies (recurrent), Dislocations (recurrent), Chronic articular pain, Chronic fatigue syndrome**
**AN_002534**	**F/39**	**-**	**+ Doughy Thin**	**+ A, H**	**+ (9/9)**	**Anisocoria, Easy bruising, Piezogenic papules, Short stature, Severe congenital kyphoscoliosis treated with surgery (arthrodesis), Bilateral valgus knees, Severe *hallux valgus*, *Pes planus*, Recurrent generalized dislocations, Vertebral dislocations treated with surgery, Hip periarthritis, Arthrosis, Tendinopathy (recurrent), Enthesopathies (recurrent), Osteoporosis, Chronic articular pain (sporadic FANS and opioids therapy), Muscle hematomas and rupture, Mitral valve prolapse, Deep venous thrombosis, Chronic fatigue syndrome, Urethral and rectal prolapse, Recurrent hemorrhagic cystitis**
**AN_002535 brother of**	**M/20**	**+**	**+ Doughy**	**+ A**	**+ (7/9)**	**Delayed anterior fontanelle closure, Epicanthus, High palate, Hypoplastic uvula, Easy bruising, Bilateral molluscoid pseudotumor (elbow), Arachnodactyly, Scoliosis, *Pectus excavatum *(marked), Toes deformities, Mobile patella, *Pes planus*, Dislocations (sporadic), Mitral valve prolapse, Chronic fatigue syndrome, Atopy, Dysphagia, Epileptic seizures**
**AN_002536 daughter of**	**F/11**	**+**	**+ Doughy**	**+ A**	**+ (7/9)**	**Blue sclerae, High palate, Micrognathia, Easy bruising, Bilateral molluscoid pseudotumor (elbow), Arachnodactyly, Mild scoliosis, *Pes planus*, Toes deformities**
**AN_002537**	**M/53**	**+**	**- Doughy**	**+ A**	**- (0/9)**	**Hypotonia at birth, Blue sclerae, Piezogenic papules, *Hallux valgus***, **Pes planus, Gastrocnemius muscle rupture, Achilles tendon rupture, Enthesopathies (left knee, recurrent), Deep venous thrombosis, Hypertension (beta-blockers treatment)**
**cEDS1**	**F/65**	**+**	**+ Loose, wrinkled**	**+ A**	**– (2/9)**	**Blue sclerae, Easy bruising, Piezogenic papules, Mild scoliosis, *Hallux valgus *treated with surgery, Subdislocations (sporadic), Chronic articular pain, Carotid stenosis in antiaggregant therapy**
**cEDS2**	**F/4**	**-**	**+ Doughy**	**+ A**	**+ (9/9)**	**Epicanthus, Blue sclerae, Anteverted nostrils, Easy bruising, Dislocations (sporadic)**
**cEDSv-l1**	**M/53**	**+**	**+ Doughy**	**+ A**	**+ (6/9)**	**Blue sclerae, High palate, Hypoplastic uvula, Easy bruising, Piezogenic papules, Molluscoid pseudotumor (elbow), *Pectus excavatum*, Severe left *hallux valgus*, *Pes planus*, Dislocations (sporadic), Chronic articular pain, Chronic perimalleolar lymphedema, Varicose veins, Mild mitral and aortic regurgitation, Left ventricular wall thickening, Aortic root ectasia, Vertebral artery tortuosity, Hepatic hemangioma, Chronic fatigue syndrome, Gastroesophageal reflux, Hiatal and abdominal hernias, Right inguinal hernia treated with surgery**

Abbreviations: *A*: atrophic scars; *H*: hypertrophic scars; *y*, years; *w*, weeks. Patient(s) ID AN_002501-37 correspond to the identifiers submitted in the LOVD EDS Variant Database (http://www.le.ac.uk/ge/collagen/); cEDS1-2: patients without identified causal mutation, not registered in LOVD; cEDSv-l1: vascular-like cEDS patient with *COL1A1* causal mutation. a: In all of the patients the blue of the sclerae was faint.

### Isolation of the DNA/RNA and mutational analysis

Genomic DNA (gDNA) was purified from blood samples of affected and unaffected family members using a Wizard Genomic DNA purification Kit (Promega, Madison, WI, USA) using standard procedures. For one patient (AN_002533), a fibroblast culture was established from a skin biopsy, as previously described [39]. The total RNA was isolated from patients’ skin fibroblasts using TRIzol reagent (Life Technologies Carlsbad, CA, USA) and was retrotranscribed in the presence of random hexanucleotide primers and MMLV-RTase (Invitrogen, Life Technologies) using standard procedures.

The primers were designed for all of the coding exons, including the intron-exon boundaries, and for the 5’ and 3’ UTRs near the coding sequence (a minimum of 200 bp). The primers were designed using a combination of the Primer3 (http://frodo.wi.mit.edu/) and PrimerZ (http://genepipe.ngc.sinica.edu.tw/primerz/) tools. The primer sequences were analyzed for the absence of known variants, such as single nucleotide polymorphisms (SNPs), using dbSNP version 135 (http://www.ncbi.nlm.nih.gov/projects/SNP/). All of the exons and intron-flanking regions of the *COL5A1*, *COL5A2* and *COL1A1* genes were amplified with standardized PCR using optimized genomic primer sets and the GoTaq Ready Mix 2X (Promega) to generate 55, 49 and 30 separate PCR fragments, respectively (primer sequences and amplification profiles are available upon request). Following the enzymatic cleanup of the PCR products by ExoSap-IT® (Affymetrix UK Ltd, Wycombe La High Wycombe, UK), all of the fragments were sequenced in both orientations using the BigDye*®* Terminator Cycle Sequencing kit protocol (Life Technologies), and Performa® DTR Ultra 96-Well Plates (EdgeBio, Gaithersburg, MD, USA) were used for PCR cleanup. This step was followed by capillary electrophoresis on the ABI3130XL Genetic Analyzer (Life Technologies). Furthermore, to verify the effect of an identified splice mutation, RT-PCR was performed using RNA that was isolated from patients’ skin fibroblasts using standard procedures. Specifically, the amplification of cDNA covering exons 36–38 of the *COL5A2* gene was performed. When a mutation was identified, the parents and unaffected family members of the proband were examined. All of the identified mutations were confirmed to not be novel polymorphisms based on the analysis of 200 alleles from healthy Italian donors and were submitted to the LOVD Ehlers–Danlos Syndrome Variant Database. The nucleotide and protein accession numbers correspond to the *COL5A1* (NM_000093.3, NP_000084.3), *COL5A2* (NM_000393.3, NP_000384.2) and *COL1A1* (NM_000088.3, NP_000079.2) reference sequences. The mutations were annotated according to HGVS nomenclature (http://www.hgvs.org/mutnomen). The nucleotide numbering is based on cDNA sequence numbering, with +1 corresponding to the A of the ATG translation initiation codon 1 in the reference sequence. For protein numbering, +1 corresponds to the first translated amino acid. The sequences were analyzed using the Sequencher 4.9 software (http://www.genecodes.com) and/or the Mutation Surveyor tool (http://www.SoftGenetics.com). The sequence descriptions from all of the mutations and polymorphisms were verified using Mutalyzer (http://www.LOVD.nl/mutalyzer). We used mutation prediction programs, such as MutationTaster (http://www.mutationtaster.org), PolyPhen-2 (http://genetics.bwh.harvard.edu/pph2/), SIFT (http://sift.jcvi.org/) and Alamut software version 2.2 (http://www.interactive-biosoftware.com), to evaluate causality based on the alteration of the protein structure. Project HOPE (http://www.cmbi.ru.nl/hope/home), a server that provides insight in the structural effects of mutations, was also used [40]. To evaluate splice site mutations, we used four prediction programs (SpliceSite-Finder-like, MaxEntScan, NNSPLICE and Human Splicing Finder) in Alamut Software.

### MLPA and SNP-array analyses

For the multiplex ligation-dependent probe amplification (MLPA), the commercially available SALSA MLPA kits P331-A1/P332-A1 for the *COL5A1* gene were used (MRC-Holland, Amsterdam, The Netherlands). The MLPA was performed in duplicate, according to the manufacturer’s recommendations (http://www.mrc-holland.com), using 100 ng of gDNA. The P331 and P332 probemixes did not contain probes for exons 12, 33, 36, 49, 54, and 66. MLPA-generated fragments were separated by capillary electrophoresis on an ABI3130XL Genetic Analyzer using 500 LIZ® as an internal size standard (Life Technologies). The results were analyzed using GeneMarker® software version 2.2.0 (SoftGenetics, State College, PA, USA). Deletions and duplications of the targeted exons were detected when the height ratios of the fluorescent peaks were lower or higher, respectively, than the normal height ratio range of 0.75-1.30.

We confirmed positive MLPA results with an Affymetrix Human Mapping GeneChip 6.0 array that contains a total of 2 million probes, half of which were polymorphic. The DNA was processed according to the manufacturer’s instructions (Affymetrix UK Ltd). To obtain intensity data, the array analysis was performed using Affymetrix GeneChip Command Console Software (AGCC). The AGCC probe cell intensity data were then analyzed using Genotype Console 3.01 (GTC3.01) to obtain genotype data. Quality thresholds were used to reduce the number of genotype errors, and the copy number (CN) state calls were generated from the BRLMM-P-Plus algorithm, which was implemented in GTC 3.0.1. This algorithm compares the intensity signal of each marker in each sample against a reference pool formed from a group of 270 samples derived from the HapMap database. Next, the software generates a median intensity value for each marker, which is reported as the log_2_ ratio of the CN state. The CN state information was then used by the Affymetrix segmentation algorithm to identify CNVs. To reduce the risk possibility of false-positive CNVs, the segmentation algorithm parameters were set to consider CNVs as only those regions larger than 100 kb, consisting of at least 25 contiguous markers without a diploid state, and with an average probe density lower than 10 kb.

## Results

### Clinical findings

**Figure 1:**
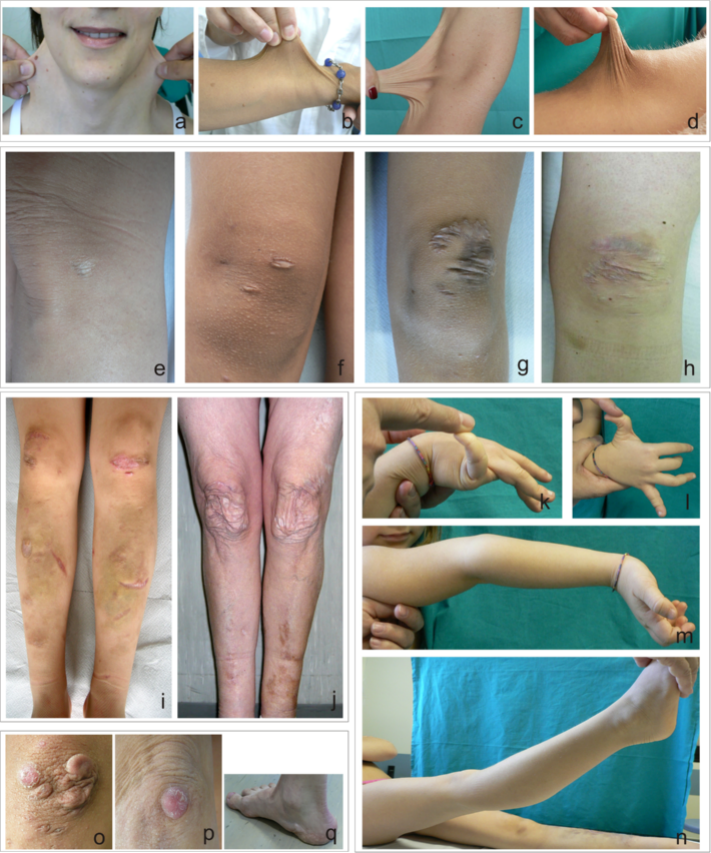
**Cutaneous and articular features in patients with cEDS. a-d)** marked skin hyperextensibility on the neck, the forearm, the elbow, and the knee; **e-h**) different scar types, small atrophic, atrophic and hypertrophic, hypertrophic and haemosiderotic; **i, j**) scars and easy bruising of the knees and pretibial area in a pediatric and an adult patient; **k-n**) hypermobility of the little finger, the thumb, the elbow and the knee in a pediatric patient; **o-p**) molluscoid pseudotumors; **q**) piezogenic papules.

A cohort of 40 patients with cEDS from 28 families were clinically evaluated (Table 1). Of the patients, there were 16 males and 24 females, 14 pediatric patients, i.e., less than 12 years of age, and 26 adults. A family history of cEDS was present in 9 probands. Patients AN_002528 and AN_002529 were 36-year-old monozygotic twin sisters. Smooth and hyperextensible skin on the neck, the forearm, the elbow and the knee was present in all of the patients to various degrees, with the only exception being patient AN_002537. In 51% of the positive patients, either pediatric or adult, the skin was markedly hyperextensible. In 18% of the patients, the skin was poorly hyperextensible (28% pediatric, and 12% adult) (Figure 1 a-d, Table 1). Skin fragility that led to defective scarring was observed in 95% of the patients (scars were absent in 2 adult females, AN_002508 and AN_002512; 2.4%) (Table 1). The scar presentations were variable with respect to type, localization, and number. Three different types of scars were observed: atrophic (small and large size, in 95% of the patients), haemosiderotic (18%, all in adults, except one), and hypertrophic (13%, primarily pediatric) (Figure 1 e-h). The sites presenting the highest incidence of scar formation were those that are most susceptible to major traumatism, i.e., the forehead (56% children and adults, with no difference between these groups), the knees (82%, in both children and adults) and the pretibial area (93% children, 76% adults) (Figure 1 i, j). A total of 50% of the pediatric patients and 35% of the adults presented with scars in all of these areas. The adults exhibited the most severe presentations. In 28% of the pediatric patients, defective scars were also observed in other sites (e.g., the chin, the elbow, and the wrist). The elbows, forearm, and thighs were affected in only 7% of the adults. Nearly all of the patients exhibited multiple aberrant scars. Only one small atrophic scar was present in two adult females (AN_002504 and AN_002511).

With respect to joint hypermobility, 32/40 patients fulfilled the Beighton score (80%) (Figure 1 k-n). In approximately 60% of the adult patients, this feature was associated with recurrent/sporadic dislocation(s)/subdislocation(s) with or without chronic or sporadic articular pain (Table 1). Only one adult male reported sporadic joint dislocations without pain (AN_002535). Four children (29% of the pediatric patients: AN_002520, AN_002521, AN_002525, and cEDS2) had events of dislocation/subdislocation, and one of these patients (AN_002520) experienced chronic pain of the knees (Table 1). None of the patients required daily therapy, except for one adult male (AN_002506), who was treated with FANS. The other patients required sporadic pain therapy with FANS. For one patient (AN_002534), who had the most severe phenotype, opioid therapy was administered following several widespread dislocations and two events of muscle rupture (Table 1).

The additional clinical manifestations included the following: dysmorphic signs (21/40 blue sclerae, 16/40 micro-retrognathia, epicanthal folds in 8/14 pediatric patients and in 6/26 adults, and 12/40 high palate); cutaneous signs (39/40 easy bruising, 26/40 doughy skin, 22/40 piezogenic papules, 5/40 molluscoid pseudotumors, 3/40 loose skin, and 3/40 *striae distensae*) (Figure 1 o-q); articular and skeletal signs (22/40 *pes planus*, 18/40 (kypho)scoliosis, 11/40 *hallux valgus*, 6/40 mild *pectus excavatum*, and 4/40 valgus knee); cardiovascular signs (7/40 valvular regurgitation, 6/40 mitral valve prolapse); and other signs (8/40 chronic fatigue syndrome, 6/40 gastroesophageal reflux; 5/40 hypotonia at birth; 4/40 hernias; and 2/40 delayed motor development) (Table 1). Spheroids were not evaluated.

**Figure 2:**
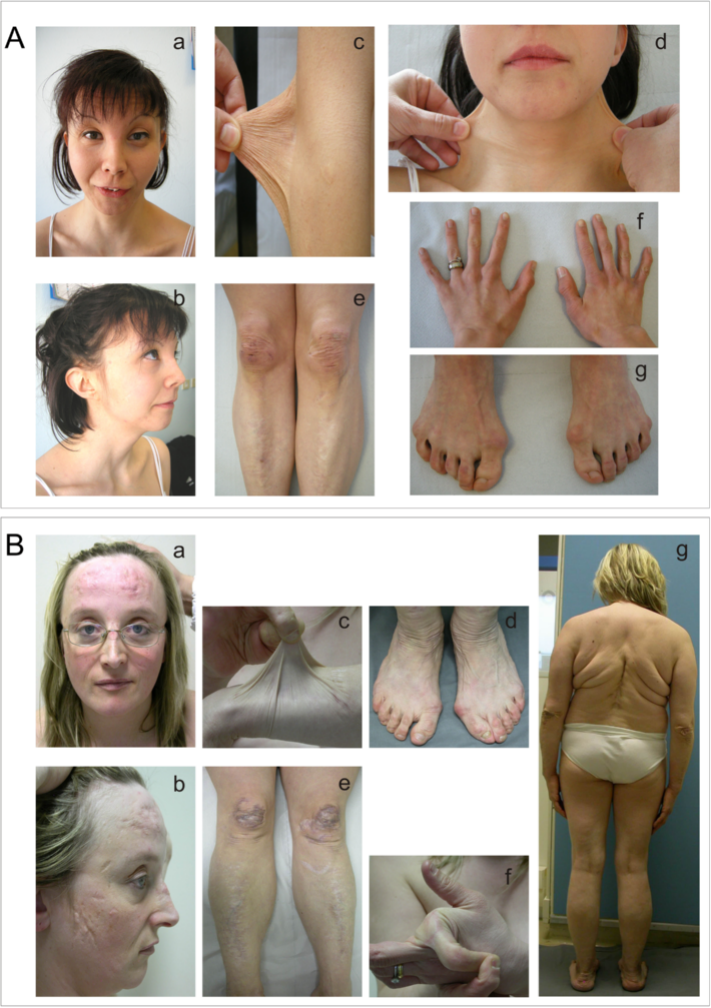
**Clinical findings in two patients with cEDS. A)** Patient AN_002502, with ocular involvement and **a, b)** dysmorphic features, including epicanthus, blue sclerae, hypertelorism, micrognathia; **c-d)** skin hyperextensibility**; e)** easy bruising and atrophic and hypertrophic scars on the knees and pretibial area; **f, g**) hand and foot deformities, *hallux valgus*. **B)** Patient AN_002534 with a severe phenotype; **a, b)** atrophic and hypertrophic scars on the forehead and the cheeks at the age of 32 years; **c**) marked skin hyperextensibility on the forearm; **d**) redundant and sagging skin on the ankles and *hallux valgus*; **e**) scars (all types) on the knees and inferior limbs; **f**) hypermobility of the forefinger; **g**) outcomes of severe congenital kyphoscoliosis treated with surgery, bilateral valgus knee, scars on the elbows, the forearms, redundant and sagging skin on the ankles, and flat feet.

Further findings were present in three subjects. Patient AN_002502 exhibited the following signs: i) ocular involvement, i,e., reduced anterior chamber depth, hypermetropia and astigmatism; ii) *facies sui generis*, with the presence of severe epicanthus, blue sclerae, hypertelorism and micrognathia; iii) skeletal abnormalities (arthrosis-like deformities of the hands and feet, scoliosis, *hallux valgus*) and spondylolisthesis; iv) hypotonia at birth with delayed motor development; and v) renal ptosis (Figure 2A, a-g; Table 1).

Patient AN_002535 presented with skeletal signs that were overlapping with Marfan syndrome, i.e., arachnodactyly, marked *pectus excavatum*, and other uncommon features of cEDS, i.e., delayed anterior fontanelle closure, epileptic seizures and hypoplastic uvula. The sister of this patient (AN_002536) also presented with arachnodactyly. The father (AN_002537) did not exhibit skeletal involvement; he experienced spontaneous gastrocnemius muscle and Achilles’ tendon rupture and exhibited recurrent enthesopathies (left knee), deep venous thrombosis, and hypertension (Table 1).

The third patient (cEDSv-l1) had more severe cardiovascular involvement than is generally observed in cEDS, i.e., aortic root ectasia, vertebral artery tortuosity, left ventricular wall thickening, mild mitral and aortic regurgitation, and hepatic hemangioma (Table 1). This patient reported two first paternal cousins with abnormal scarring who were both dead at 32 years from cerebral aneurism rupture; their mother exhibited abnormal scarring and died at 70 years from an unspecified vascular disease.

Patients AN_002533 and AN_002534 were 21 and 39 years old, respectively, and presented an extremely severe phenotype, including failure to thrive, marked involvement of the cutaneous and musculoskeletal systems, chronic pain, and chronic fatigue syndrome (Table 1). Specifically, the 39-year-old patient (AN_002534) exhibited severe congenital kyphoscoliosis and vertebral dislocations that were both treated with surgery, hip periarthritis, osteoporosis, arthrosis, short stature (1.41 m, below the 3^rd^ percentile), rectal and urethral prolapse, deep venous thrombosis, two events of muscle rupture, and hematomas (Figure 2B, a-g).

During childhood, cutaneous involvement was the most prominent sign in the majority of the patients; the phenotype worsened in the teenage years and adulthood with various degrees of musculoskeletal and systemic features. Neither severe aneurysmatic dilatation nor arterial rupture were present.

Wide intrafamilial phenotypic heterogeneity was observed and was most remarkable in AN_002507 and AN_002513 patients’ mothers, who were apparently unaffected based on the absence of scars and for whom the only presence of musculoskeletal signs did not suggest the presence of the disorder until cEDS was diagnosed for their children (Table 1). Patient AN_002537 differed primarily from his two affected children in that he did not exhibit skin hyperextensibility and joint hypermobility (Table 1).

The phenotypes of AN_002528 and AN_002529, who were twin sisters, were nearly completely overlapping and showed signs of a full presentation of the disorder (Table 1).

### Molecular characterization and genotype-phenotype correlations

**Figure 3:**
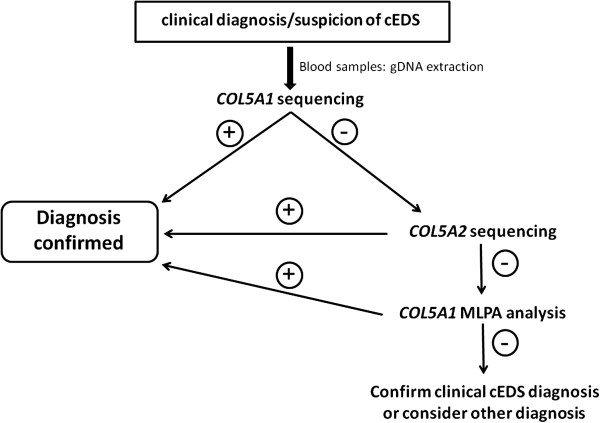
**Diagnostic flowchart adopted in this study.** The diagnostic strategy was based on the clinical evaluation of the patients followed by *COL5A1* sequencing, which detected the causal mutation in the large majority of the cases. *COL5A2* was investigated when *COL5A1* analysis was negative; MLPA analysis was performed when the two previous analyses were not able to detect the causal mutation.

The flowchart that was adopted for mutation detection in the examined patients with cEDS began with direct sequencing of the *COL5A1* gene. If no mutation was detected, *COL5A2* was analyzed (Figure 3). In patients without defects, the presence of large genomic rearrangements in the *COL5A1* gene was investigated by MLPA, which is not commercially available for *COL5A2* (Figure 3). In patients without a detected mutation but with a clear phenotype, the clinical diagnosis of cEDS was maintained. Other diagnoses were considered for patients with additional signs. For example, one patient (cEDSv-l1) had a family history of cardiovascular involvement (Table 1), and we suspected vascular-like cEDS. This patient’s *COL1A1* gene was sequenced and was found to contain the previously described c.934C>T mutation. This mutation results in an arginine-to-cysteine substitution p.(Arg312Cys) in the α1(I)-collagen chain at the Xaa-position of the repeating structure of the triple helical domain.

Using this flowchart, we were able to identify the causal mutations in our cEDS patient cohort with a detection rate of approximately 93% (26/28 characterized probands). Of the identified *COL5A* mutations, 91% involved the *COL5A1* gene. Of these, 18 were novel mutations, 2 of which were detected twice in the present study (c.2988del and c.3769C>T), and 3 were previously reported (c.2988dup, c.3413G>A and c.3769C>T) [18; LOVD]. Two novel splice mutations were detected in the *COL5A2* gene (Table 2). Parental testing indicated that 14 of the 23 *COL5A* mutations arose *de novo*; for 2 mutations, *de novo* testing was not possible as the parents were unavailable, but family history was reported to be negative. Eight mutations were detected in families with multiple affected members (Table 2). In two patients (cEDS1 and cEDS2), no causal mutations in the *COL5A1*, *COL5A2* genes were detected, although these patients did not differ clinically from the mutation-positive patients (Table 1).

**Table 2:** ***COL5A1 *and *COL5A2 *mutations in patients with cEDS**

**Patient(s) IDs**	**Gene**	**Exon, intron/localization**	^**a **^**Change at the nucleotide level**	^**b **^**Nonsense/Missense**	^**b **^**Frameshift**	**Splice site**
**Mutations leading to type V collagen haploinsufficiency**						
**AN_002501**	**A1**	**ex1/N-propeptide**	**c.87G>A°**	p.(Trp29*)		
**AN_002503-05#**	**A1**	**int7/N-propeptide**	**c.1165-2A>G**		p.(Pro389Leufs*168)	^**c **^**Activation of cryptic splice acceptor site 4 bp downstream of WT acceptor**
**AN_002506**	**A1**	**ex13/N-propeptide**	**c.1651C>T°**	p.(Gln551*)		
**AN_002509**	**A1**	**int31/helix**	**c.2647-12A>G°**		p.(Gly883Leufs*195)	^**c **^**Creation of new splice acceptor site 11 bp upstream of WT acceptor**
**AN_002510**	**A1**	**ex34/helix**	**c.2757_2774del18insA°**		p.(Glu920Hisfs*14)	
**AN_002511**	**A1**	**ex36/helix**	**c.2891dup°**		p.(Gly967Trpfs*47)	
AN_002514-15*	**A1**	**ex38/helix**	**c.2988del°**		p.(Gly997Alafs*77)	
**AN_002516**	**A1**	**ex38/helix**	**c.2988dup^ **^**e**^		p.(Gly997Argfs*17)	
**AN_002517**	**A1**	**ex42/helix**	**c.3328C>T°**	p.(Gln1110*)		
**AN_002520-23#**	**A1**	**ex45/helix**	**c.3568C>T**	p.(Gln1190*)		
AN_002524-25*	**A1**	**ex48/helix**	**c.3769C>T° **^**e**^	p.(Arg1257*)		
**AN_002527**	**A1**	**ex62/C-propeptide**	**c.4714del°**		p.(Val1572Serfs*47)	
**AN_002528-29#**	**A1**	**ex62/C-propeptide**	**c.4919_4928del10^**		p.(Lys1640Serfs*86)	
**AN_002530-31#**	**A1**	**ex63/C-propeptide**	**c.4962C>G**	p.(Tyr1654*)		
**AN_002532**	**A1**	**ex66/C-propeptide**	**c.5458_5459del°**		p.(Phe1820Argfs*2)	
**Large genomic rearrangement identified by MLPA and SNP-array**						
**AN_002535-37#**	**A1**		**chr9.hg19:g.(137,440,166_137,442,686)_(137,633,699_137,638,368)dup**			
**Mutations affecting the structural integrity of type V collagen**						
**AN_002502**	**A1**	**ex4/N-propeptide**	**c.532A>C°**	**p.(Thr178Pro)**		
**AN_002507-08#**	**A1**	**ex29/helix**	**c.2436A>T**	**p.(Glu812Asp)**		^**c **^**alteration of an ESE sequence, splice error?**
**AN_002512-13#**	**A1**	**int37/helix**	**c.2952+2_2952+3del**	**p.(Gly967_Thr984del)**		^**c **^**in-frame exon 37 skipping**
**AN_002518-19#**	**A1**	**ex43/helix**	**c.3413G>A**^**e**^	**p.(Gly1138Glu)**		
**AN_002526**	**A1**	**ex54/helix**	**c.4178G>A°**	**p.(Gly1393Asp)**		
**AN_002533**	**A2**	**ex29/helix**	**c.1977G>A°**	**p.(Gly642_Pro659del)**		^**c **^**in-frame exon 29 skipping**
**AN_002534**	**A2**	**int37/helix**	**c.2499+2T>C°**	**p.(Gly816_Pro833del)**		^**d **^**in-frame exon 37 skipping**

The patient(s) IDs correspond to the identifiers found in the LOVD EDS Variant Database (http://www.le.ac.uk/ge/collagen/); ^a^ DNA mutation numbering is based on the cDNA sequence. For cDNA numbering, +1 corresponds to the A of the ATG translation initiation codon in the reference sequence. The reference sequences are based on the GenBank Accession no.: *COL5A1* NM_000093.3, *COL5A2* NM_000393.3. ^b^ For protein numbering, +1 corresponds to the first translated amino acid. The reference sequences are based on GenBank Accession no.: *COL5A1* NP_000084.3, *COL5A2* NP_000384.2. ^c^ The effect on splicing was analyzed using four prediction programs (SpliceSite-Finder-like, MaxEntScan, NNSPLICE and Human Splicing Finder) in Alamut software, version 2.2 (Additional file 1: Figures S1–S6). ^d^ The effect on mRNA splicing was verified by RT-PCR of total RNA that was purified from the patient’s skin fibroblasts (Additional file 1: Figure S6B). ° *de novo* mutation verified by parental testing. ^ Parents not available for *de novo* testing. # Members of the same family. * Members of different families. ^e^ Mutation previously reported [18, LOVD].

Among the 21 *COL5A1* mutations that were identified in the present study, approximately 76% were null allele mutations that were predicted to cause COLLV haploinsufficiency given that they should activate the NMD pathway (Table 2). Specifically, 6 were nonsense mutations and 7 were small genomic deletions/duplications that caused frameshift and the formation of a PTC. Among these mutations, two affected the c.2988 position and were present in three unrelated patients. Patients AN_002514 and AN_002515 (both c.2988del), as well as AN_002516 (c.2988dup), presented a complete phenotype that was compatible with their age (Table 1). Patients AN_002524 and AN_002525, who carried the c.3769C>T transition [p.(Arg1257*)], presented a complete and mild phenotype, respectively, according to their age (Table 1).

Two mRNA splicing errors that were predicted to cause haploinsufficiency were also detected (Table 2). We identified a c.1165-2A>G splice acceptor mutation in intron 7 in the AN_002503-05’s family. This mutation is predicted *in silico* to cause the activation of a cryptic splice acceptor site 4 bp downstream of the canonical site inside of exon 8, therefore generating a PTC [p.(Pro389Leufs*168)] (Additional file 1: Figure S1). However, a more complex splicing outcome cannot be excluded; for example, in-frame exon skipping of one or more exons, as observed for the previously reported c.655-2A>G, c.925-2A>G, and c.925-1G>C mutations [18,34,35], is possible. In this case this mutation should be considered to be structural. In patient AN_002509, the *de novo* c.2647-12A>G mutation in intron 31 was predicted *in silico* to create a new splice acceptor site 11 bp upstream of the consensus acceptor site, with the retention of the last 11 bases of intron 31, a frameshift and PTC formation [p.(Gly883Leufs*195)] (Additional file 1: Figure S2).

**Figure 4:**
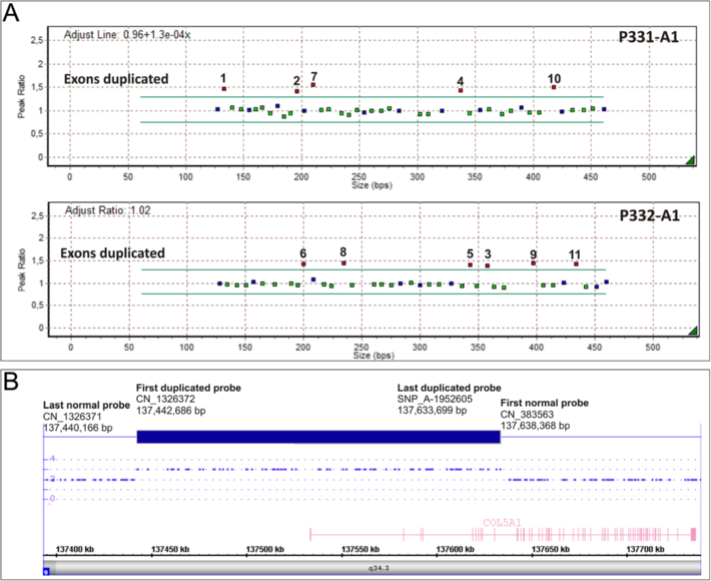
**Large genomic duplication in the *COL5A1 *gene. A**) MLPA analysis showed the duplication of exons 1–11 of the *COL5A1* gene that was identified using the SALSA MLPA kits P331-A1 (upper panel) and P332-A1 (lower panel). The MLPA results were analyzed using GeneMarker® software. **B)** Affymetrix Human Mapping GeneChip 6.0 array analysis, which was performed to define the duplication size, revealed an approximately 191 kb duplication, including the *COL5A1* proximal promoter region: chr9.hg19:g.(137,440,166_137,442,686)_(137,633,699_137,638,368)dup. The last normal probe (CN_1326371), the first duplicated probe (CN_1326372) at the 5’ end, the last duplicated probe (SNP_A-1952605), and the first normal probe (CN_383563) at the 3’ end define the duplicated area.

In patient AN_002535, for whom *COL5A1* and *COL5A2* mutations were not observed based on genomic sequencing, a large genomic duplication encompassing exons 1–11 was identified by MLPA (Figure 4A). The presence of the duplication was confirmed by SNP-array analysis, and its size and boundaries were defined, revealing an approximately 191 kb duplication [chr9.hg19:g.(137,440,166_137,442,686)_(137,633,699_137,638,368)dup] that included the *COL5A1* proximal promoter region (Figure 4B, Table 2). The duplication was inherited from the proband’s affected father (AN_002537) and was also identified in the patient’s affected sister (AN_002536 in Table 1). Although the precise break point and orientation of the duplication was not examined, this mutation likely causes an aberrant out-of-frame mRNA splicing and consequent haploinsufficiency.

The patients who harbored mutations that led to haploinsufficiency generally presented complete phenotypes, even with a given degree of age-dependent and inter/intrafamilial variability. An exception to this was patient AN_002537, who did not meet two of the major criteria of the disorder (Table 1). Only a single scar each was observed for patients AN_002504 and AN_002511, who were both young adults. In patient AN_002511, that only one scar was present this was likely due to constant skin care and the avoidance of traumatisms since childhood. Patient AN_002535, who carried the large genomic duplication in *COL5A1*, exhibited skeletal signs of Marfan syndrome and other unusual signs. His 11-year-old sister (AN_002536) only presented arachnodactyly, whereas their father (AN_002537) presented with none of these signs (Table 1).

Only approximately 24% (5/21) of the *COL5A1* mutations, i.e., four missense and one splice error, were identified to affect the structural integrity of the α1(V)-chain (Table 2). We identified a missense mutation in the TSPN-1 domain of the α1(V)-N-propeptide in patient AN_002502. This *de novo* c.532A>C mutation in exon 4 replaced the highly conserved threonine in position 178 with a proline [p.(Thr178Pro)]. The wild type residue is predicted to form hydrogen bonds with the threonine at position 188 and with the asparagine at position 176. The difference in hydrophobicity should affect this hydrogen bond formation, the 3D-structure, and the function of the TSPN-1 domain. This mutation leads to a multisystem severe phenotype that includes ocular involvement (Table 1, Figure 2A).

We detected the c.2436A>T mutation in exon 29 in the AN_002507-08 family. This mutation replaces the glutamic acid at position 812 with an aspartic acid [p.(Glu812Asp)]. This highly conserved residue is located at the X position of the G-Xaa-Yaa motif in the triple helix domain; therefore, the mutation is predicted to interfere with the triple helix structural integrity. This variant is categorized by Polyphen2 as probably damaging and by SIFT as deleterious. Several bioinformatics tools suggest that this mutation alters exonic splicing enhancer (ESE) sequences, i.e., the SF2/ASF binding site AAGACGG shows a decreased consensus score (3.1 vs. 1.98 by ESE-finder), and the GTGAAG and the GAAGAC consensus sites are abolished (Rescue_ESE). Therefore, an aberrant splicing outcome, e.g., in-frame exon 29 skipping, cannot be excluded (Additional file 1: Figure S3). The AN_002507 pediatric patient exhibited the three major cEDS criteria, certain dysmorphisms and easy bruising without other signs and complications. In contrast, her mother (patient AN_002508) did not fulfill the major criteria and was diagnosed following the characterization of her daughter (Table 1).

Lastly, in the AN_002512-13 family, a small genomic deletion (c.2952+2_2952+3del) that involved the splice donor site of exon 37 in the α1(V)-triple helix domain was detected. This mutation was *in silico* predicted to cause in-frame skipping of exon 37 [p.(Gly967_Thr984del)] (Additional file 1: Figure S4). The activation of a cryptic splice donor site within intron 37 cannot be excluded; in this case, the mutation’s consequence would most likely be frameshift and NMD. Patient AN_002513 showed a complete phenotype, whereas his 41-year-old mother (AN_002512) did not and was diagnosed following the characterization of her son (Table 1).

The c.3413G>A (exon 43) and c.4178G>A (exon 54) mutations were identified in the AN_002518-19 family and in AN_002526, respectively. These are typical glycine substitutions within the triple helix domain that result in replacement of glycine with a bulkier amino acid [p.(Gly1138Glu) and p.(Gly1393Asp)]. Patients AN_002518 and AN_002519 showed a complete phenotype; AN_002526 was the most severely affected patient among those carrying a *COL5A1* mutation (Table 1).

In conclusion, considering all of the characterized patients with *COL5A1* mutations, genotype-phenotype correlations were not observed when comparing the phenotypic severity of patients with a null allele and those with structural mutations. One exception to this is a possible correlation between a glycine substitution in the C-terminus of the collagenous domain and a severe phenotype, as was observed for patient AN_002526. Furthermore, the structural mutation in the N-terminal region of the pro-α1(V) collagen chain in patient AN_002502 was associated with unusual ocular involvement and a severe phenotype.

The two patients of our cohort with the most severe phenotype were determined to carry *COL5A2* defects. In both of these patients, a structural mutation appeared *de novo* and produced in-frame skipping of the affected exon. Specifically, for patient AN_002533 (c.1977G>A), a synonymous substitution in the codon for the proline at position 659 was detected. This mutation alters the consensus donor splice site and is predicted *in silico* to cause the in-frame deletion of the 18 amino acids that are encoded by exon 29 [p.(Gly642_Pro659del)] (Additional file 1: Figure S5). The skipping of exon 29 was also reported for the c.1947A>G transition within this exon, abolishing an ESE consensus site [18]. The second *COL5A2* mRNA splice mutation was identified in patient AN_002534 and consisted of a c. 2499+2T>C transition, which affects the consensus donor splice site of exon 37 and is predicted to cause in-frame exon skipping (Additional file 1: Figure S6A). This prediction was verified by performing RT-PCR of total RNA that was purified from the patient’s skin fibroblasts; this analysis confirmed the in-frame skipping of exon 37 [p.(Gly816_Pro833del)] (Additional file 1: Figure S6B).

## Discussion

cEDS is a rare multisystem disorder that is incompletely understood at the clinical and molecular level. In this 3-year work, we adopted a diagnostic strategy that was based on a careful clinical evaluation of 40 patients followed by a specific molecular analysis flowchart developed to simplify and expedite the diagnostic procedure. The physical examination revealed high inter- and intra-familial variability in cEDS, with phenotypes ranging from severe to nearly undetectable. The molecular analysis allowed for the identification of *COL5A1/COL5A2* defects in approximately 93% of the probands, supporting the recent findings that these are the major, if not the only, causal genes of cEDS [18].

The clinical diagnosis of cEDS was based on Villefranche nosology’s major and minor criteria, including cutaneous involvement, i.e., skin hyperextensibility, defective scarring, and articular hypermobility [1-6,18]. We observed that skin hyperextensibility was present to a varying degree, ranging from marked to poor or even absent. Poorly hypextensible skin is often present in hEDS patients, who exhibit a certain degree of clinical overlap with cEDS, especially with those without marked skin hyperextensibility [10]. Multiple abnormal scars were observed in 95% of the patients. Only a small atrophic scar was present in two adult patients, and scars were not detected in two patients. The absence of atrophic scars or the presence of atrophic post-surgical scars is often observed in hEDS patients [10, our observations]. Therefore, the above mentioned patients, who also presented articular involvement and minor signs that are common to hEDS, had a phenotype that was compatible with hEDS. The diagnosis of cEDS was reached based on the existence of relatives who were clearly affected with cEDS, i.e., fulfilling the three major criteria, and was confirmed using molecular genetics testing that identified the causal mutations. The third major criterion of cEDS, i.e., joint hypermobility, was present in all but eight of the patients. These observations confirm that, in our cohort, the three major Villefranche criteria are useful and sufficient for a clinical diagnosis of cEDS in the large majority of patients [18]. These criteria are inadequate for a small number of subjects, but minor signs and family history can suggest the presence of the disorder also in patients who do not fulfill the three major criteria. In the absence of family history, patients without clear skin involvement and joint hypermobility are not easily diagnosed by physicians and can even escape clinical observation; therefore, the disorder is most likely under-recognized and is certainly underestimated. For all of the borderline patients, a reliable genetic test can rapidly make a diagnosis or suggest a diagnosis of hEDS or other connective tissue disorders. Minor criteria affect, to varying degrees, the cutaneous, articular and musculoskeletal systems and include both dysmorphic and systemic signs. Such signs support the clinical diagnosis and confirm generalized connective tissue fragility/involvement. More specifically, musculoskeletal involvement and articular chronic pain, either localized or generalized, was observed in 60% of the adult patients. Interestingly, none of these patients used daily therapy, except for one patient who was treated with FANS. The other patients required pain therapy with FANS for sporadic events. The greater tolerance of articular pain and the different pain therapies distinguishes cEDS from hEDS patients, with the latter in general requiring daily treatment, often with opioid drugs [41].

Molecular analyses of *COL5A1* and *COL5A2* genes detected defects in 26 of 28 cEDS probands (approximately 93%), adding 20 novel mutations to those that have been previously reported. More than 60% of these mutations arose *de novo*, and nearly all were private mutations, except for two *COL5A*1 mutations that were identified twice in the present study and three *COL5A1* mutations that were reported previously [18, and LOVD].

The majority of the causal mutations affected the *COL5A1* gene (90%), which is in agreement with the data that are reported in the LOVD EDS variant database. Similarly, approximately two-thirds of these *COL5A1* mutations were null allele mutations, comprising nonsense, small del/ins, splice errors and a large duplication. All of these mutations led to COLLV haploinsufficiency. Of these, the most notable is the 191 kb duplication involving the *COL5A1* proximal promoter and the first 11 exons. This case is the second gross genomic rearrangement that has been described to affect this gene [20].

Only five structural mutations were identified in *COL5A1*: two typical glycine substitutions in the triple helix domain, two other unusual missense mutations, and one in-frame exon skipping mutation. Of the unusual missense mutations, one was within the TSPN-1 domain of the α1(V)-N-propeptide, and the second was at the X position of the Gly-Xaa-Yaa motif, most likely resulting in altered splicing. Among structural mutations, the most notable is p.(Thr178Pro), which affects the TSPN-1 domain. This mutation is the first missense mutation that has been identified in the α1(V)-N-propeptide and is predicted to disturb both conformation and function, with a dominant negative effect of perturbing collagen fibrillogenesis and ECM organization. Indeed, the three other reported mutations that affect the α1(V)-N-propeptide are known to i) interfere with the formation of COLLV heterotrimers and of the heterotypic fibrils and ii) disturb the interactions between COLLV and other ECM components, such as type VI collagen, TGF-β1, and PCPE-1 (Procollagen-C-Proteinase Enhancer 1) [16,18,34,35].

Only two splice mutations were detected in *COL5A2*; this low frequency and the type of mutation, i.e., in-frame skipping of the affected exons with a dominant negative effect, are in agreement with the data for this gene deposited in the LOVD.

Lastly, the *COL1A1* mutation p.(Arg312Cys), which is known to be involved in the vascular-like cEDS [36], was detected in one patient. Apart from the vascular involvement, this patient did not show features different from those observed in our cEDS patient’s cohort.

To date, no clear genotype-phenotype correlations have been established for cEDS. This lack of correlations is hypothesized to be related to the fact that COLLV is a minor component of heterotypic fibrils that primarily contain COLLI. This latter protein is much more abundant in the ECM than COLLV and primarily has a structural support function. In contrast, COLLV has a regulatory role in collagen fibrillogenesis. It therefore was hypothesized that the perturbation of this regulatory function, irrespective of the nature of the COLLV mutation, is the principal determinant of the functional and phenotypic outcome. This situation suggests a common pathogenic pathway for the majority of COLLV mutations, in which the reduced availability of wild type COLLV is crucial. However, in certain cases, other factors, such as increased ER stress and/or disturbed interactions with other ECM molecules, could also contribute to the phenotypic outcome [18,32-35]. In this view, certain exceptions can be drawn for a subset of mutations. Specifically, mutations with an atypical splicing outcome in the N-terminal-propeptide domain of α1(V) appear to be associated with a severe phenotype [18,34,35], as do the two *COL5A1* glycine substitutions [p.(Gly493Arg) and p.(Gly1564Asp)] that were observed in patients with cEDS with vascular complications [9,18]. The severe multi-systemic phenotype with ocular involvement of the patient with the mutation in the α1(V)-N-propeptide domain strengthens the hypothesis that alterations in this domain lead to severe clinical presentation. Interestingly, the patient who was reported by Symoens et al. also exhibited unusual eye involvement [35]. Furthermore, given that, among the identified *COL5A1* mutations, the p.(Gly1393Asp) missense mutation at the C-terminus of the collagenous domain was associated with the most severe phenotype, we can speculate that the rare *COL5A1* glycine substitutions can also lead to a severe phenotype, even without vascular complications. In contrast, given that patients with mutations leading to *COL5A1* haploinsufficiency can also present with a severe phenotype, even with arterial rupture, other genetic and environmental factors likely modulate cEDS phenotypes [4,8,18]. Although the number of clinically well-described patients with cEDS and *COL5A2* mutations is limited, the majority of them have severe phenotypes, as in this study, and all of the *COL5A2* mutations were structural with a dominant negative effect [4,18,30,32]. This observation may therefore represent a consistent genotype-phenotype correlation. Lastly, our patients and their relatives who carried the *COL1A1* p.(Arg312Cys) mutation typically showed a cEDS phenotype with severe vascular involvement and a propensity for arterial rupture in young adulthood [36,38]. In general, genotype-phenotype correlations are not evident in cEDS, and different genetic backgrounds involving possible modulator genes and environmental factors likely contribute to the wide inter- and intra-familial variability irrespective of the mutation type. Although the number of patients who have been characterized at a clinical and molecular level is limited, certain exceptions still emerge that may be useful for genetic counseling. The identical clinical presentation of the two monozygotic twin sisters that were characterized in this study support the above-mentioned role of modulator genes and environment in cEDS clinical presentation. Moreover, concerning intrafamilial variability, the most remarkable phenotypic differences that we observed were in two mothers and their children (families AN_002507-08 and AN_002512-13). These patients carried missense mutations with a possible splice effect and a donor splice mutation; we therefore speculate that these mutations can lead to different intrafamilial outcomes due to the individual set and efficiency of splicing factors that mature the aberrant mRNAs, thus explaining the different clinical severities. More specifically, in family AN_002507-08, the p.(Glu812Asp) mutation can be a simple missense or could lead to the in-frame skipping of exon 29; likewise, in family AN_002512-13, the detected donor splice mutation may lead to structural in-frame skipping of exon 37 or may activate a cryptic splice donor site with consequent haploinsufficiency.

Here, we adopted a diagnostic cEDS flowchart that was based principally on the clinical evaluation of the patients and direct sequencing of the major genes that are involved, *COL5A1* and *COL5A2.* We began with DNA that was purified from whole blood samples. To date DNA sequencing has become a time-saving and cost-effective approach compared to other techniques, even including pre-screening methods, such as DHPLC and HRM analyses. In addition to sequencing, we included *COL5A1* MLPA analysis to detect possible gross rearrangements. Using this strategy, we failed to identify the causal mutation for only two patients who had a clear cEDS phenotype. Although additional genetic heterogeneity cannot be entirely excluded, the lack of identified mutations is most likely the result of technical limitations of the sequencing method; for example, the mutation detection strategy does not allow for the identification of deep intronic mutations, mutations in the regulatory regions (promoter, 3’UTR, etc.), or large genomic alterations in *COL5A2*. This limitation is because MLPA analysis is unavailable for this gene and because of rare private variants that fall within the primer targeted sequences that could avoid PCR amplification of the mutant allele.

Our molecular approach was selected to avoid the requirement for a skin biopsy for diagnostic purposes. This was done for the following reasons: i) biopsies are invasive procedures that are not readily accepted by patients with defective wound healing and for whom a clear clinical diagnosis can be reached; ii) the establishment of *in vitro* fibroblast cultures requires specific technical facilities and skills and is both costly and time-consuming; iii) the identification of the causal mutation in the *COL5A1* gene beginning with cDNA is not possible for null allele mutations, which represent the most frequent alterations in this gene. The *COL5A1* null allele test still must be considered to be a valid approach for the evaluation of the presence of a causal mutation; however, it does not provide the type and position of the mutation, which is detected by gDNA sequencing. The null allele test may have been useful in the case of our two patients without an identified causal mutation. Furthermore, skin biopsy was not considered for COLLV biochemical analyses as it allows for the detection of abnormal protein in only approximately 10% of patients [4,18].

Although our diagnostic strategy was very effective, certain drawbacks remain. Primary among these was the impossibility of examining the outcome of splice mutations at the RNA level or the effect of given missense mutations at the protein level. This problem is relevant primarily for basic research and for understanding the genotype-phenotype correlation; however, such information is not useful to the diagnostic procedure. The identification of the causal mutation in patients with cEDS remains a milestone with respect to the diagnostic *iter* for these patients, especially for patients with unclear clinical presentation. Moreover, the identification of these mutations allows for effective genetic counseling, follow-up and prenatal diagnosis.

In conclusion, this study indicates that the accurate clinical evaluation of the patients and their relatives reduces the number of doubtful diagnoses. Using the flowchart here adopted, the patients without a clear suspect of cEDS can be ascertained, and diagnoses can be confirmed by genetic testing, allowing for the identification of the majority of the molecular defects.

### Consent

Written informed consent was obtained from patients for publication of this research article and accompanying images. A copy of the written consent is available for review by the Editor-in-Chief of this journal.

## Abbreviations

cEDS: Classic Ehlers–Danlos syndrome; cDNA: Complementary DNA; CNV: Copy number variation; COLLI: Type I collagen; COLLV: Type V collagen; DHPLC: Denaturing high-pressure liquid chromatography; ECM: Extracellular matrix; gDNA: Genomic DNA; hEDS: Hypermobility type Ehlers–Danlos syndrome; HRM: High-resolution melting; MLPA: Multiple ligation-dependent probe amplification; NMD: Nonsense-mediated RNA decay; PTC: Premature termination codon of translation; RT-PCR: Reverse transcriptase-polymerase chain reaction; SNP: Single nucleotide polymorphism; TSPN-1: N-terminal thrombospondin-1-like.

## Competing interests

The authors declare that they have no competing interest.

## Authors’ contributions

MR and MC conceived of the study. MV, CD, AV, AW, LG, EM, PCP, and MC recruited and made the clinical diagnoses of the patients and performed the genetic counseling and follow-up. MR, NC, SQ, MT performed the molecular analyses. MR, CD, and NC researched the literature, reviewed and prepared the manuscript. NZ prepared the fibroblast cultures. MC and MR edited and coordinated the manuscript. All of the authors discussed, read, and approved the manuscript.

## Supplementary Material

Additional file 1: Figure S1 *In silico* prediction of the effect of the c.1165-2A>G splice acceptor mutation (intron 7, *COL5A1*) in the AN_002503-05 patient’s family using four prediction programs (SpliceSite-Finder-like, MaxEntScan, NNSPLICE and Human Splicing Finder) in Alamut Software version 2.2. This mutation is predicted to cause the activation of a cryptic splice acceptor site 4 bp downstream of the canonical site inside exon 8, generating a frameshift and PTC formation [p.(Pro389Leufs*168)]. **Figure S2.** In patient AN_002509, the *de novo* c.2647-12A>G mutation in intron 31 of *COL5A1* is predicted to create a new splice acceptor site 11 bp upstream of the consensus acceptor site, with the retention of the last 11 bases of intron 31 and a consequent frameshift and PTC formation [p.(Gly883Leufs*195)]. **Figure S3.***In silico* analysis of the c.2436A>T mutation [p.(Glu812Asp)] in exon 29 of *COL5A1* in the AN_002507-08 family suggests that this transversion alters exonic splicing enhancer (ESE) sequences. The SF2/ASF binding site AAGACGG has a decreased consensus score (3.1 vs. 1.98 by ESE-finder), and the GTGAAG and GAAGAC consensus sites are abolished (Rescue_ESE). Therefore, an aberrant splicing outcome, for example in-frame skipping of exon 29, cannot be excluded. **Figure S4.** The small genomic deletion (c.2952+2_2952+3del), which abolishes the splice donor site of exon 37 of *COL5A1*, was detected in the AN_002512-13 family and is predicted to cause in-frame skipping of exon 37 p.(Gly967_Thr984del). **Figure S5.** The *COL5A2* transition c.1977G>A in patient AN_002533. This mutation is a synonymous substitution in the codon of the proline at position 659 and abolishes the consensus donor splice site. It is therefore predicted that this mutation causes the in-frame deletion of the 18 amino acids that are encoded by exon 29 p.(Gly642_Pro659del). **Figure S6. A.** The *COL5A2* transition c.2499+2T>C in patient AN_002534 affects the consensus donor splice site of exon 37 and is predicted to cause in-frame skipping of exon 37. **B.** RT-PCR of the total RNA that was purified from patient AN_002534’s skin fibroblasts using primers encompassing exons 36-38. The results demonstrate that the c.2499+2T>C mutation leads to in-frame exon 37 skipping [p.(Gly816_Pro833del)]. Click here for file
