# Supplementary material for: Clinical and molecular characterization of 40 patients with classic Ehlers–Danlos syndrome: identification of 18 COL5A1 and 2 COL5A2 novel mutations
Source: Orphanet J Rare Dis. 2013 Apr 12;8:58. doi: 10.1186/1750-1172-8-58 (PMC3653713; doi:10.1186/1750-1172-8-58)

**ADDITIONAL DATA**

**Additional Figure 1.** *In silico* prediction of the effect of the c.1165-2A>G splice acceptor mutation (intron 7, *COL5A1*) in the AN_002503-05 patient’s family using four prediction programs (SpliceSite-Finder-like, MaxEntScan, NNSPLICE and Human Splicing Finder) in Alamut Software version 2.2. This mutation is predicted to cause the activation of a cryptic splice acceptor site 4 bp downstream of the canonical site inside exon 8, generating a frameshift and PTC formation [p.(Pro389Leufs*168)].


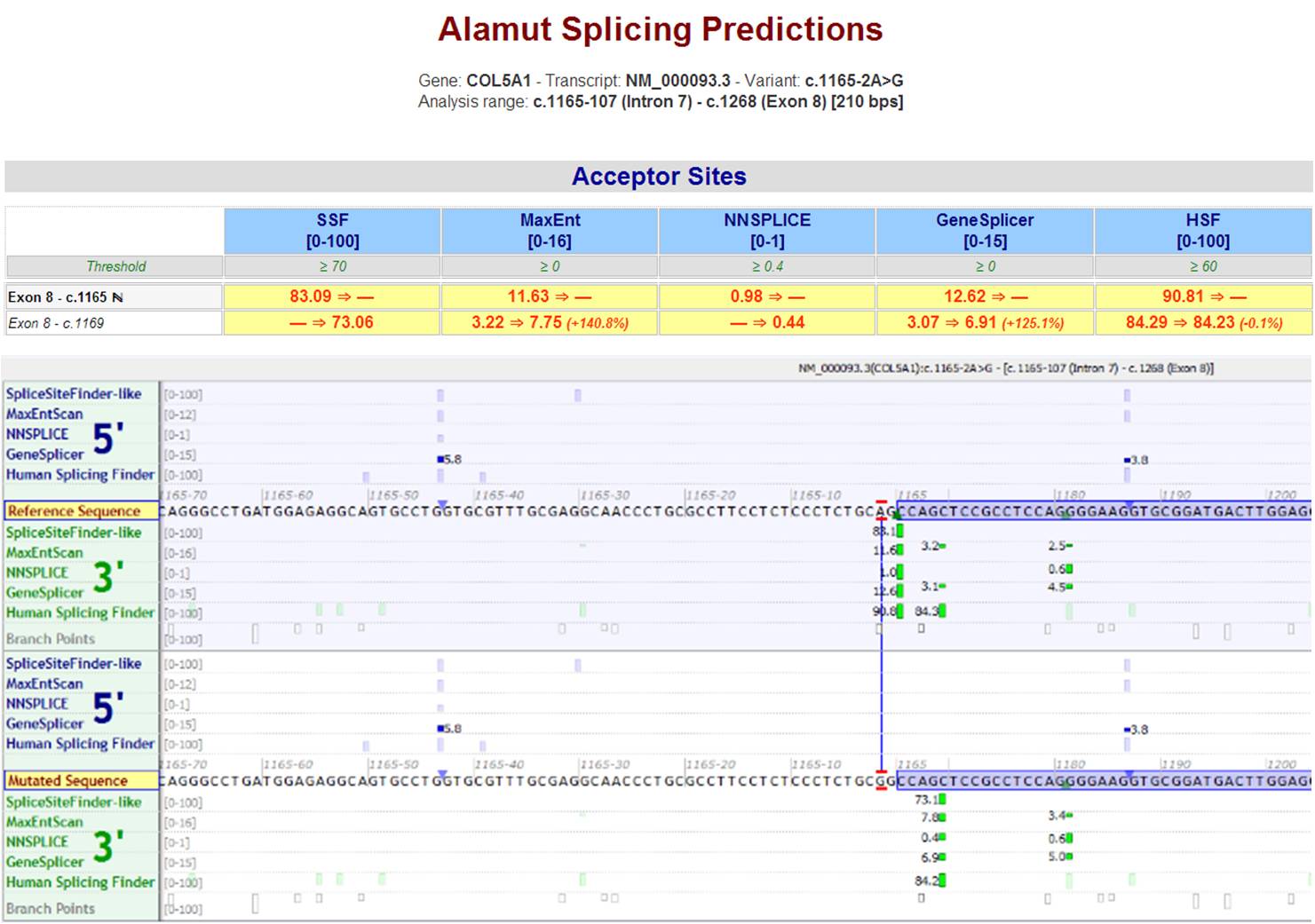


**Additional Figure 2.** In patient AN_002509, the *de novo* c.2647-12A>G mutation in intron 31 of *COL5A1* is predicted to create a new splice acceptor site 11 bp upstream of the consensus acceptor site, with the retention of the last 11 bases of intron 31 and a consequent frameshift and PTC formation [p.(Gly883Leufs*195)].

**
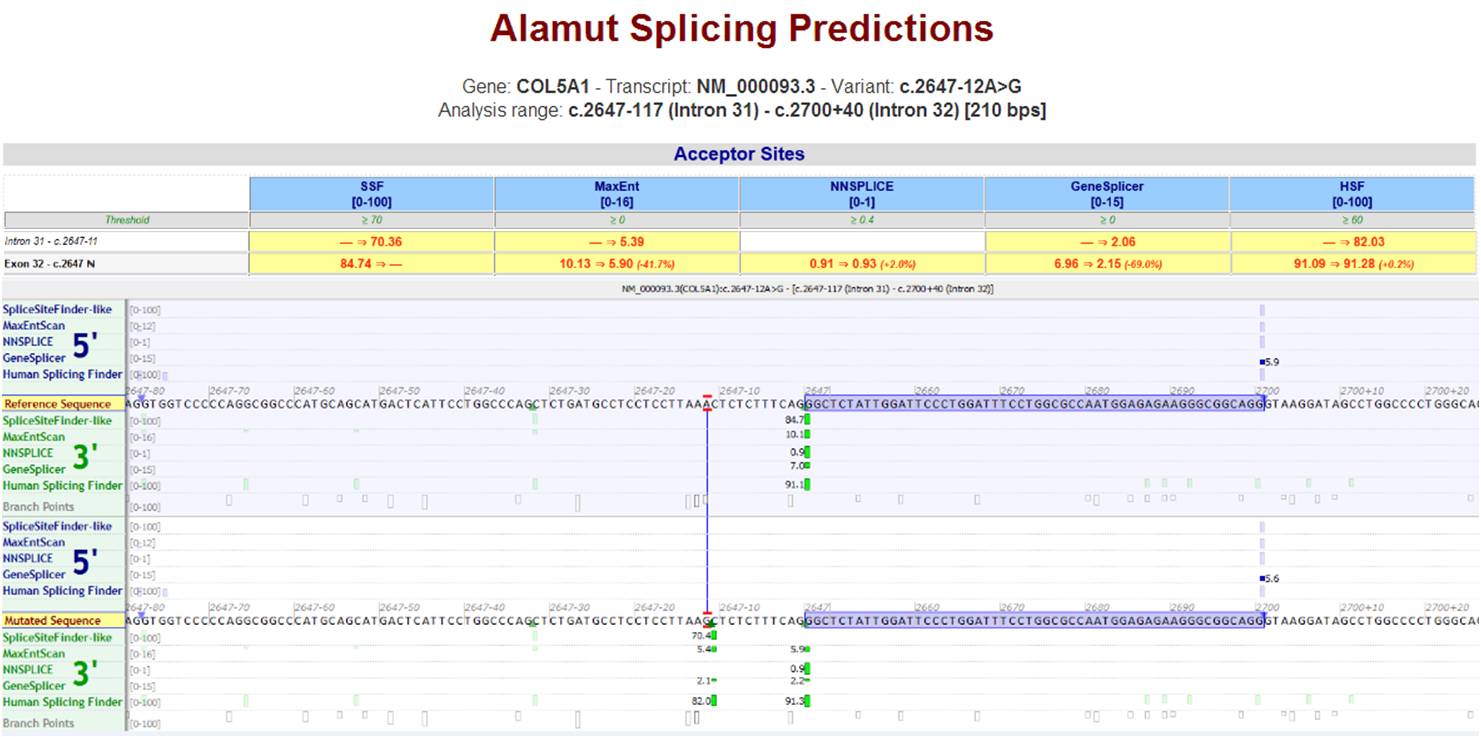
**

**Additional Figure 3.** *In silico* analysis of the c.2436A>T mutation [p.(Glu812Asp)] in exon 29 of *COL5A1* in the AN_002507-08 family suggests that this transversion alters exonic splicing enhancer (ESE) sequences. The SF2/ASF binding site AAGACGG has a decreased consensus score (3.1 vs. 1.98 by ESE-finder), and the GTGAAG and GAAGAC consensus sites are abolished (Rescue_ESE). Therefore, an aberrant splicing outcome, for example in-frame skipping of exon 29, cannot be excluded.


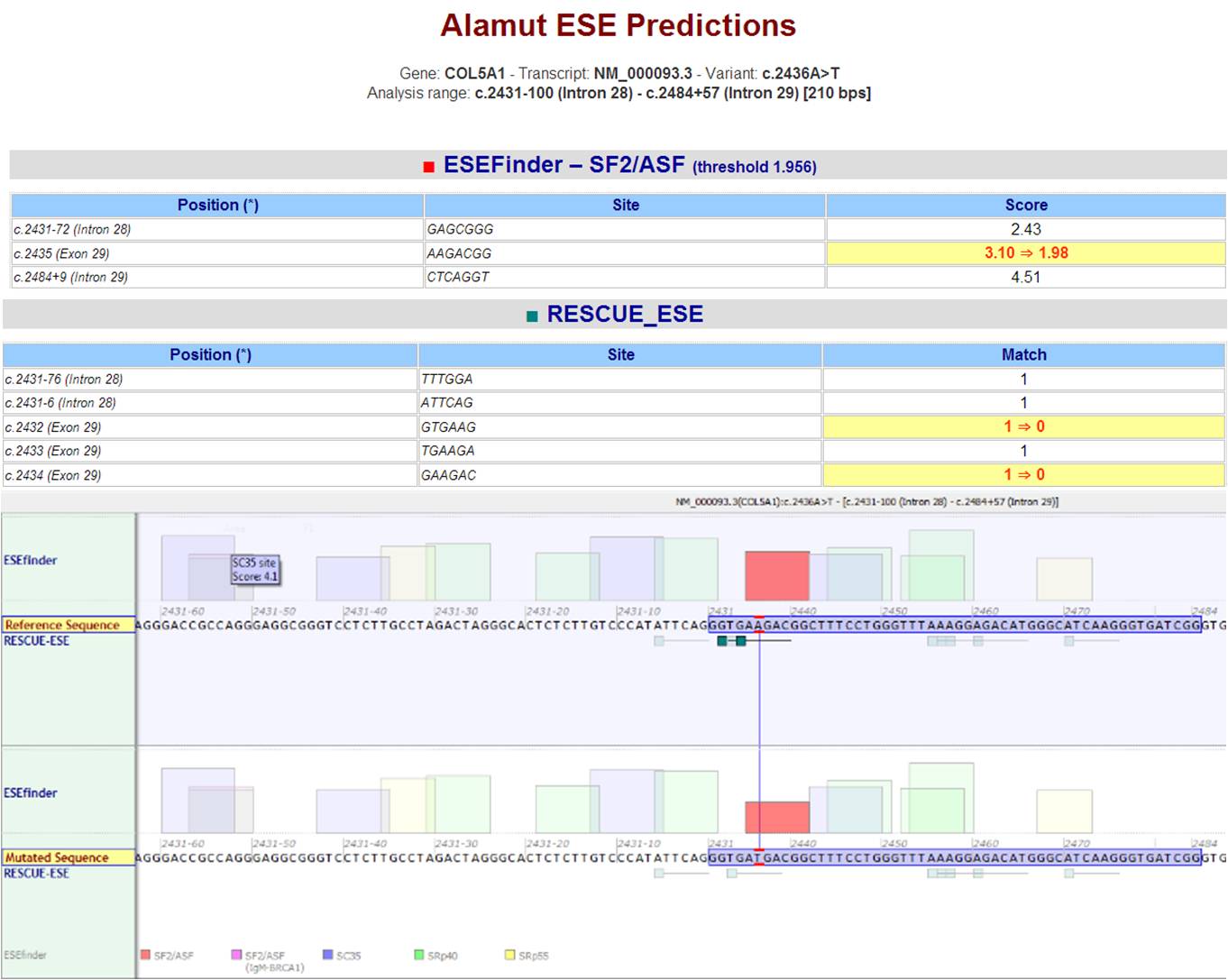


**Additional Figure 4.** The small genomic deletion (c.2952+2_2952+3delTG), which abolishes the splice donor site of exon 37 of *COL5A1*, was detected in the AN_002512-13 family and is predicted to cause in-frame skipping of exon 37 p.(Gly967_Thr984del).


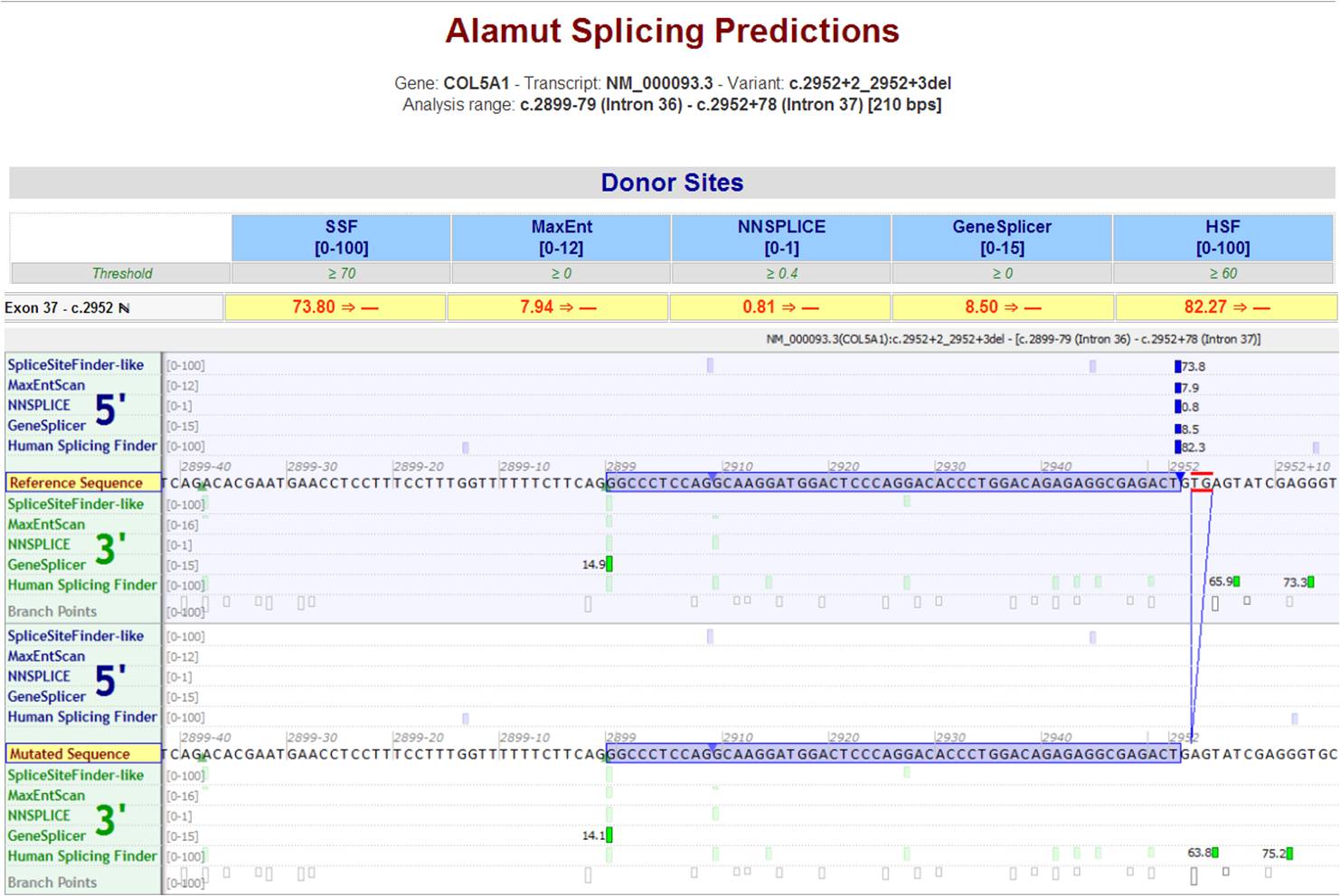


**Additional Figure 5.** The *COL5A2* transition c.1977G>A in patient AN_002533. This mutation is a synonymous substitution in the codon of the proline at position 659 and abolishes the consensus donor splice site. It is therefore predicted that this mutation causes the in-frame deletion of the 18 amino acids that are encoded by exon 29 p.(Gly642_Pro659del).


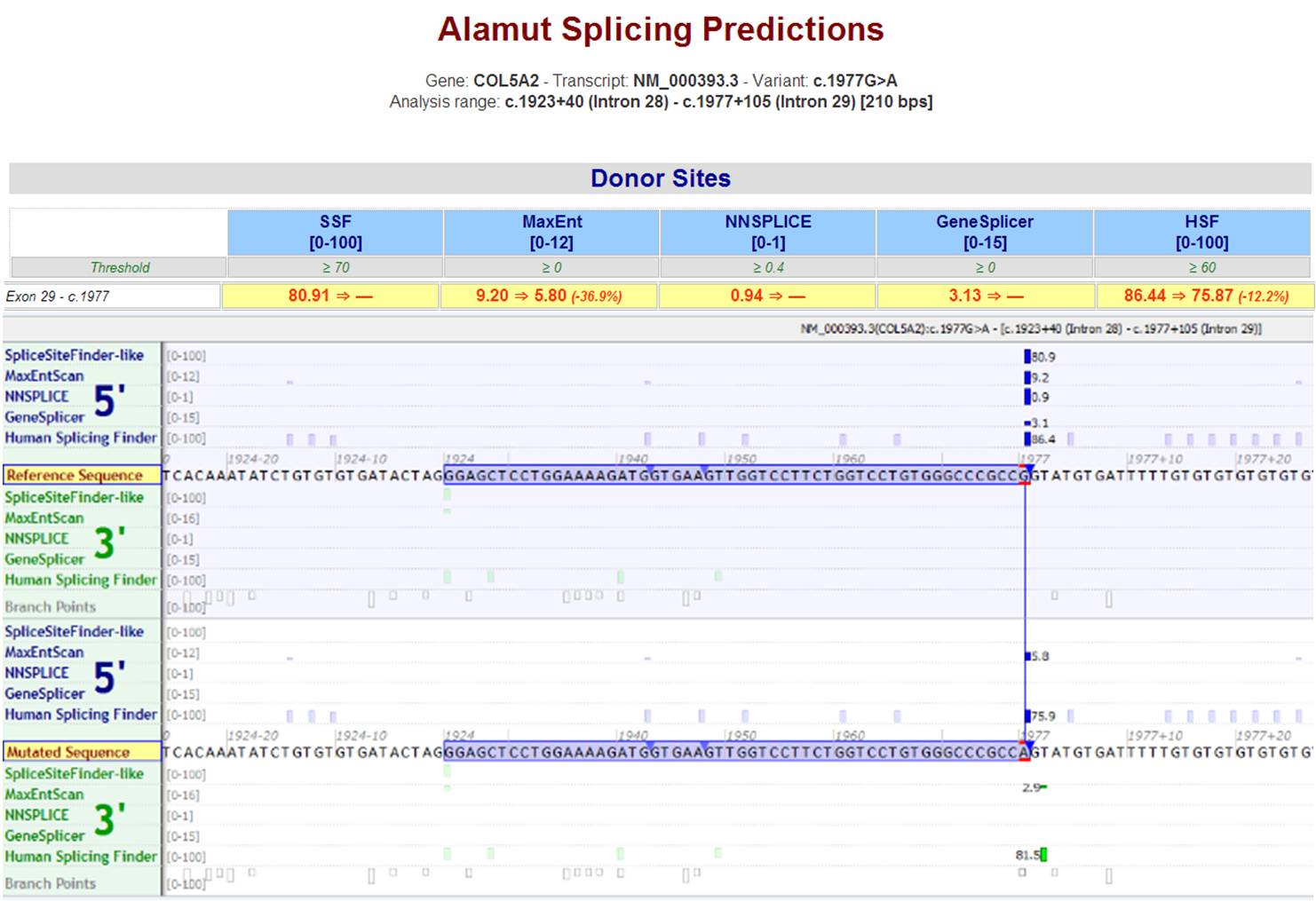


**Additional Figure 6A.** The *COL5A2* transition c.2499+2T>C in patient AN_002534 affects the consensus donor splice site of exon 37 and is predicted to cause in-frame skipping of exon 37.

**
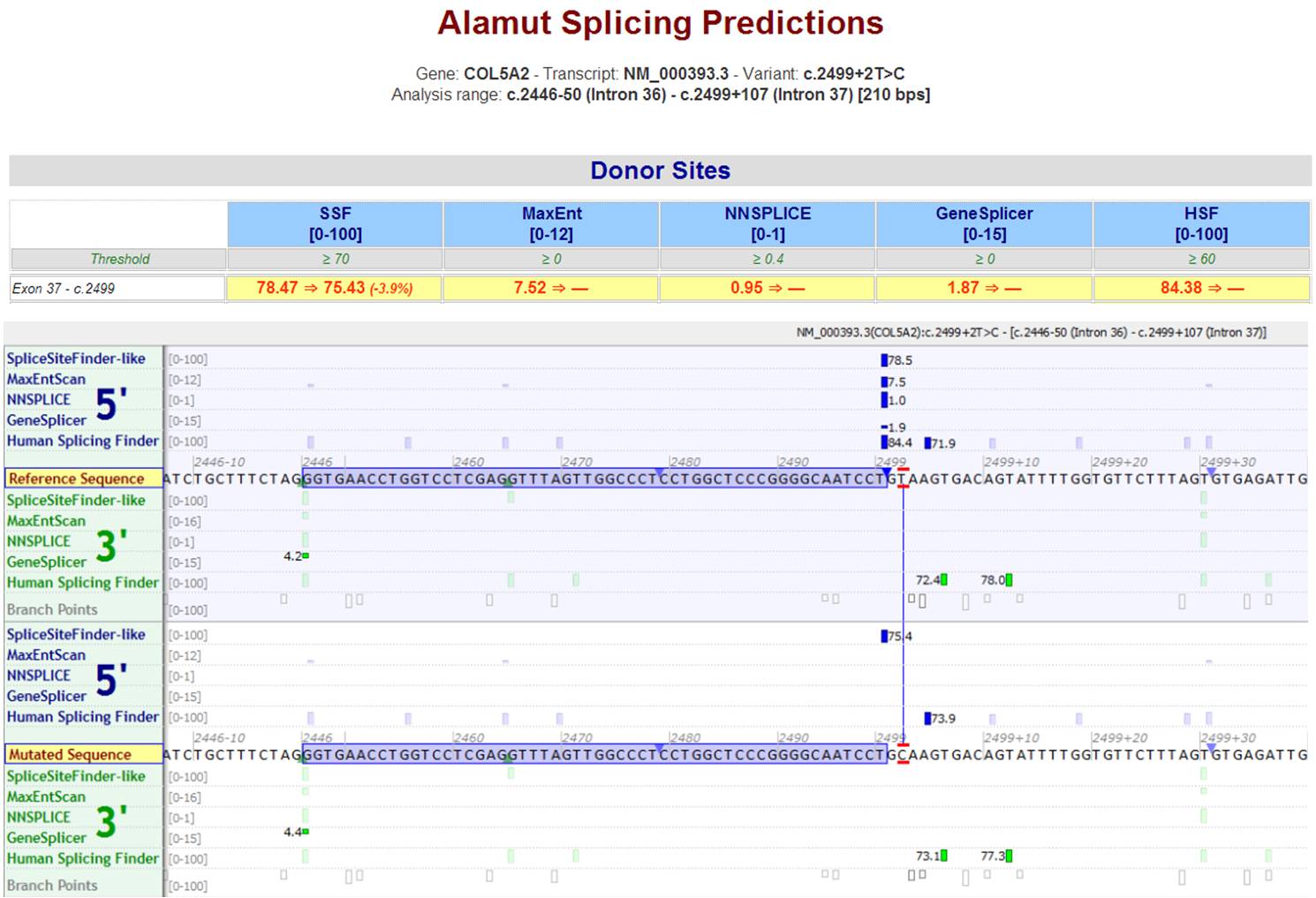
**

**Additional Figure 6B.** RT-PCR of the total RNA that was purified from patient AN_002534’s skin fibroblasts using primers encompassing exons 36-38. The results demonstrate that the c.2499+2T>C mutation leads to in-frame exon 37 skipping [p.(Gly816_Pro833del)].


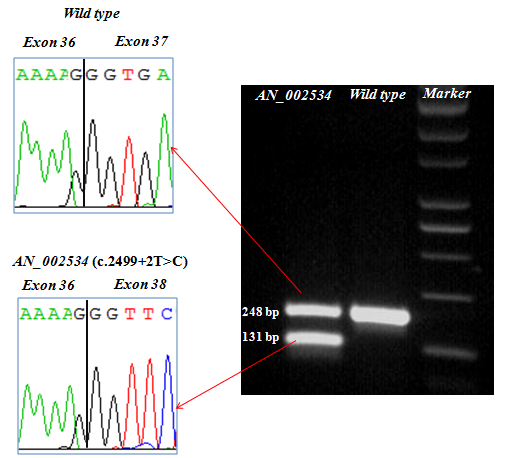

Supplement: Additional file 1: Figure S1 — In silico prediction of the effect of the c.1165-2A>G splice acceptor mutation (intron 7, COL5A1) in the AN_002503-05 patient’s family using four prediction programs (SpliceSite-Finder-like, MaxEntScan, NNSPLICE and Human Splicing Finder) in Alamut Software version 2.2. This mutation is predicted to cause the activation of a cryptic splice acceptor site 4 bp downstream of the canonical site inside exon 8, generating a frameshift and PTC formation [p.(Pro389Leufs*168)]. Figure S2. In patient AN_002509, the de novo c.2647-12A>G mutation in intron 31 of COL5A1 is predicted to create a new splice acceptor site 11 bp upstream of the consensus acceptor site, with the retention of the last 11 bases of intron 31 and a consequent frameshift and PTC formation [p.(Gly883Leufs*195)]. Figure S3.In silico analysis of the c.2436A>T mutation [p.(Glu812Asp)] in exon 29 of COL5A1 in the AN_002507-08 family suggests that this transversion alters exonic splicing enhancer (ESE) sequences. The SF2/ASF binding site AAGACGG has a decreased consensus score (3.1 vs. 1.98 by ESE-finder), and the GTGAAG and GAAGAC consensus sites are abolished (Rescue_ESE). Therefore, an aberrant splicing outcome, for example in-frame skipping of exon 29, cannot be excluded. Figure S4. The small genomic deletion (c.2952+2_2952+3del), which abolishes the splice donor site of exon 37 of COL5A1, was detected in the AN_002512-13 family and is predicted to cause in-frame skipping of exon 37 p.(Gly967_Thr984del). Figure S5. The COL5A2 transition c.1977G>A in patient AN_002533. This mutation is a synonymous substitution in the codon of the proline at position 659 and abolishes the consensus donor splice site. It is therefore predicted that this mutation causes the in-frame deletion of the 18 amino acids that are encoded by exon 29 p.(Gly642_Pro659del). Figure S6. A. The COL5A2 transition c.2499+2T>C in patient AN_002534 affects the consensus donor splice site of exon 37 and is predicted to cause in-frame skipping of [file 1750-1172-8-58-S1.doc]
